# Targeted gold-coated iron oxide nanoparticles for CD163 detection in atherosclerosis by MRI

**DOI:** 10.1038/srep17135

**Published:** 2015-11-30

**Authors:** Carlos Tarin, Monica Carril, Jose Luis Martin-Ventura, Irati Markuerkiaga, Daniel Padro, Patricia Llamas-Granda, Juan Antonio Moreno, Isabel García, Nuria Genicio, Sandra Plaza-Garcia, Luis Miguel Blanco-Colio, Soledad Penades, Jesus Egido

**Affiliations:** 1 Laboratorio de Patología Vascular y Renal. IIS Fundación Jiménez Díaz, Universidad Autónoma. Av. Reyes Católicos 2, 28040, Madrid, Spain; 2 Laboratorio de Gliconanotecnología. Biofunctional Nanomaterials Unit. CIC biomaGUNE. Paseo Miramón, 182, 20009, San Sebastián, Spain; 3 Ikerbasque, Basque Foundation for Science, 48011, Bilbao, Spain; 4 Molecular Imaging Unit, CIC biomaGUNE, PaseoMiramón 182, 20009, San Sebastián, Spain; 5 Biomedical Research Networking Center in Bioengineering, Biomaterials and Nanomedicine (CIBER-BBN), Paseo Miramón 182, 20009, San Sebastián, Spain

## Abstract

CD163 is a membrane receptor expressed by macrophage lineage. Studies performed in atherosclerosis have shown that CD163 expression is increased at inflammatory sites, pointing at the presence of intraplaque hemorrhagic sites or asymptomatic plaques. Hence, imaging of CD163 expressing macrophages is an interesting strategy in order to detect atherosclerotic plaques. We have prepared a targeted probe based on gold-coated iron oxide nanoparticles vectorized with an anti-CD163 antibody for the specific detection of CD163 by MRI. Firstly, the specificity of the targeted probe was validated *in vitro* by incubation of the probe with CD163(+) or (−) macrophages. The probe was able to selectively detect CD163(+) macrophages both in human and murine cells. Subsequently, the targeted probe was injected in 16 weeks old apoE deficient mice developing atherosclerotic lesions and the pararenal abdominal aorta was imaged by MRI. The accumulation of probe in the site of interest increased over time and the signal intensity decreased significantly 48 hours after the injection. Hence, we have developed a highly sensitive targeted probe capable of detecting CD163-expressing macrophages that could provide useful information about the state of the atheromatous lesions.

Atherosclerosis is a complex disease that involves chronic inflammation and remodelling processes that may lead to the stenosis of the aorta. It can slowly progress over decades but under certain circumstances, it can either grow quickly or destabilize, leading to an ischemic event^1^. Its high morbidity and associated mortality have made atherothrombosis one of the leading causes of death in the developed world. Consequently, a large amount of resources are spent every year trying to improve its diagnosis, prognosis and treatment with the purpose of preventing a fatal event^1^,^2^.

CD163 is a membrane receptor which functions primarily as a hemoglobin (Hb) scavenger receptor, removing the complex hemoglobin-haptoglobin from the plasma and avoiding the toxic effects of free Hb. The role of CD163 has been widely studied and linked with inflammation through its function of removing pro-inflammatory ligands such as the TNF superfamily member, TWEAK^3^. It has been described that CD163 is expressed by the monocytic-macrophage lineage, although monocytes have a modest expression level that is increased when they acquire macrophage characteristics^3^. Macrophages are one of the main inflammatory cells that play key role in the development and progression of atheroma plaque^4^. Studies performed in atherosclerosis have shown that CD163 is expressed by anti-inflammatory M2-macrophages instead of pro-inflammatory M1-macrophages and its expression is increased at inflammatory sites, specially in plaques at sites of hemorrhage^5^,^6^ or in asymptomatic plaques^7^ while healthy aortas do not contain or contain few CD163 (+) cells^8^,^9^.

Magnetic resonance imaging (MRI) has gained relevance in cardiovascular pathology detection because of its good spatial resolution and average contrast agent sensitivity^10^. The development of new contrast agents that provide more than an imaging enhancement is one of the main hot topics in MRI development nowadays. In the field of contrast enhanced imaging of atherosclerosis, the detection of the plaque presence is no longer enough and efforts have been focused on providing improved information, in a non-invasive way, about the molecular composition and/or the state of the plaque. Targeting of macrophages (usually total population or M1 fraction) with T_1_ or T_2_ contrast agents is a frequent strategy for atherosclerosis imaging by magnetic resonance^11^, either by passive labelling^12^,^13^,^14^ or by active targeting of membrane receptors with vectorized probes^15^,^16^,^17^,^18^,^19^,^20^. Nano-sized superparamagnetic iron oxide probes have been previously explored as T_2_ contrast agents for atheroma plaque imaging^12^,^13^,^14^,^15^,^21^. Among them, gold-coated iron oxide nanoparticles (NP) are particularly stable and versatile platforms. The gold coating protects the magnetite core from oxidation and allows for easy and practical functionalization of the nanoparticle surface by using thiol-ending ligands^22^. Indeed, gold-coated iron oxide nanoparticles vectorized with antibodies have been successfully prepared and validated as contrast agents both *in vitro* and *in vivo* in the targeting of different types of cells^23^,^24^,^25^. Herein, we prepared gold-coated iron oxide nanoparticles functionalized with antibodies against CD163 as a T_2_ contrast agent for the detection of M2-macrophages. Such probe was able to selectively bind to CD163-expressing macrophages *in vitro* in human and murine cells, as well as *in vivo* in a murine model of atherosclerosis. We demonstrated the ability of our probe to detect CD163 in the pathological tissue, as well as its versatility to be vectorized with immunoglobulin-G type (IgG) antibodies of our election^23^,^24^,^25^.

## Results

### Characterization of nanoparticles

**Figure 1:**
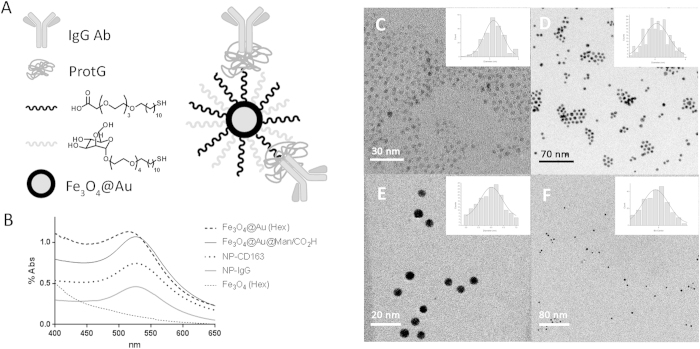
Characterization of glyconanoparticles. (**A**) Schematic representation of the nanoparticles. (**B**) UV-Vis spectra of all nanoparticles showing the appearance of the characteristic plasmon of gold. Due to their different solubilities, Fe_3_O_4_ NPs and Fe_3_O_4_@Au NPs were measured in hexane solution, whereas the rest were measured in water. (**C**) TEM micrograph and histogram of Fe_3_O_4_ NPs. (**D**) TEM micrograph and histogram of Fe_3_O_4_@Au NPs. (**E**) TEM micrograph and histogram of Fe_3_O_4_@Au@Man/CO_2_H NPs. (**F**) TEM micrograph and histogram of NP-CD163.

The prepared nanoparticles consisted of a gold-coated iron oxide core covered with thiol ligands bearing either a mannose or a carboxylic acid. ProtG was covalently linked through a peptide bond to the carboxylic moieties and IgG antibodies subsequently grafted onto them (Fig. 1A). Bradford analysis of the unbound protG or antibody found in each step of the functionalization showed that the amount of protG-IgG complex on each nanoparticle ranged from 1 to 2 units. Further evidence of the grafting of the antibodies on the nanoparticles was obtained by SDS-PAGE gels of digested nanoparticles which showed the typical 2 bands of IgG antibodies, at 25 KDa and 50 KDa for the light and heavy chains, respectively (See supplemental Figure S4)^23^. TEM micrographs showed that the average diameter of iron oxide core was 3.2 nm, which increased up to 6.1 nm after the gold coating process (Fig. 1C–F). UV-Vis spectra were measured and as expected the characteristic plasmon resonance band of gold at around 520 nm appeared in all nanoparticles after the gold coating process (Fig. 1B). Gold and iron content was obtained by ICP-OES (Inductively coupled plasma - optical emission spectroscopy) analysis on Fe_3_O_4_@Au@Man/CO_2_H NPs and it was found to be 59.3 ± 0.8% and 4.03 ± 0.05% in weight, respectively. Relaxivity measurements were performed at 11.7 T and room temperature resulting in *r*_2_ and *r*_1_ values of 160 mM^−1^s^−1^ and 10 mM^−1^s^−1^, respectively. The full characterization of these nanoparticles, as superparamagnetic materials and contrast agents, has been previously described by our group^22^,^23^,^24^,^25^,^26^.

As part of the characterization of the NPs cell viability, toxicological profile, excretion and biodistribution studies were performed using NP-CD163(m). Cytotoxicity was not detected on RAW264.7 cell cultures incubated with increasing amounts of the tested probe (See supplemental Figure S5). In addition, the biochemical profiles of the control and the injected groups and were very similar. Urea and blood urea nitrogen levels were decreased at 24 hours after NP-CD163(m) injection, showing a better kidney function. Alkaline phosphatase activity (AP) was increased at 24 hours after injection but other metabolic liver enzymes and related metabolites such as bilirubin were similar to the control group. Also this increase of AP activity diminished to normal range at 48 hours post-injection (See supplemental Table S1). In addition, histopathological analysis was performed comparing tissue sections (stained with hematoxylin-eosin) between the control and injected groups no differences were observed (See supplemental Figure S6). Post-mortem gold content analysis of main organs by ICP-OES 48 hours after injection showed that NP-CD163(m) accumulated mainly in the liver (below 10% of the injected dose), followed in decreasing order by accumulation in spleen, lung and kidney (less than 0.5% of the injected dose for each tissue). At that time point, NPs were not detected neither in serum nor in urine by ICP-OES. Aorta of ApoE mice that have not developed atheroma plaques was also examined showing non-detectable accumulation of probe (See supplemental Figure S7). Gold deposits were detected by gold staining in tissue sections obtained from several organs, which was in agreement with the results obtained by ICP-OES (See supplemental Figure S8).

### Binding of nanoparticles to CD163, *in vitro* models

**Figure 2:**
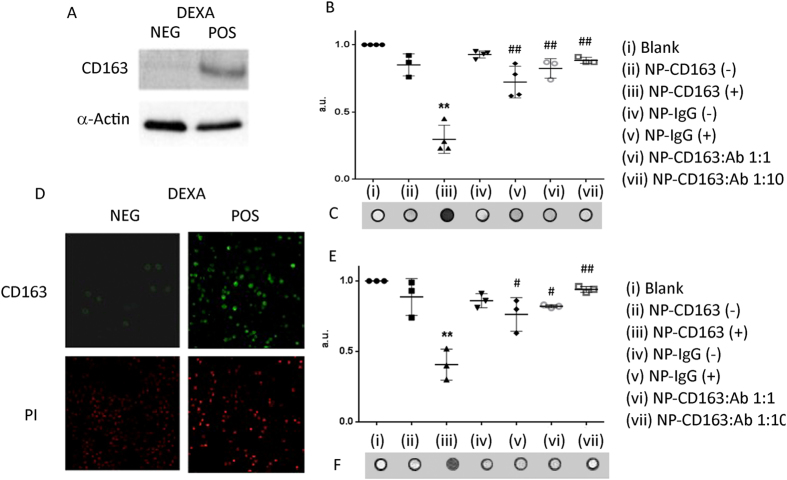
CD163 detection in *in vitro* models. (**A**) Western blot of CD163 expression and α-actin (loading control) in THP-1 macrophages cell lysates with or without dexamethasone treatment for 24 hours. (**B**) Graph showing the normalized T_2_ values obtained from MRI images of human macrophages CD163 (+) and CD163 (−) incubated with NP-CD163(h) and NP-IgG(h), n  = 3. All the replicates were normalized to the blank (cells that have not been incubated with any nanoparticles). The error bars shows the SEM. $p < 0.05 *versus* Blank; *p < 0.05, **p < 0.01 and ***p < 0.001 *versus* NP-CD163(+) (**C**) Representative MRI phantoms of human macrophages CD163 (+) and CD163 (−) incubated with NP-CD163(h) and NP-IgG(h). (**D**) Immunodetection of CD163 expression in murine macrophages with or without dexamethasone treatment. Propidium iodide (PI) was used for nuclei staining. (**E**) Graph showing the normalized mean value obtained from MRI images of murine macrophages CD163 (+) and CD163 (−) incubated with NP-CD163(m) and NP-IgG(m), n = 3. All the replicates were normalized to the blank (cells that have not been incubated with any nanoparticles). The error bars shows the SEM. ^$$^p < 0.01 *versus* Blank; *p < 0.05 and **p < 0.01 *versus* NP-CD163(+); ^#^p < 0.05 *versus* Ab 1:1. **F**) Representative MRI phantoms of murine macrophages CD163 (+) and CD163 (−) incubated with NP-CD163(m) and NP-IgG(m).

The incubation of cells that expressed (+) or not (−) CD163 (Fig. 2A,D) with the targeted or control probes showed that the NPs bearing the anti-CD163 antibody could selectively detect CD163 expressing macrophages (Fig. 2B-C, E-F). In both human and murine CD163 (+) cells incubated with NP-CD163, there was a significant decrease in the T_2_ values with respect to CD163 (−) macrophages incubated with the same probe (p < 0.05 either THP-1 or mouse peritoneal macrophages) (See Supplementary tables S1-S6 for statistical details). Likewise, T_2_ values were significantly lower than those for the same cells incubated with the NP-IgG control probe compared with NP-CD163 (p < 0.001 in THP-1 cell line and p < 0.01 in mouse peritoneal macrophages). Incubation with increasing amounts of free antibody blocked the targeting of CD163 by the NP-CD163 probe with a T_2_ values similar to that of cells not expressing CD163 or those that were incubated with control probe. This fact showed the specificity of the vectorized probe to target CD163 and excluded unspecific signal due to the probe.

### *In vivo* CD163 detection in a murine model

**Figure 3:**
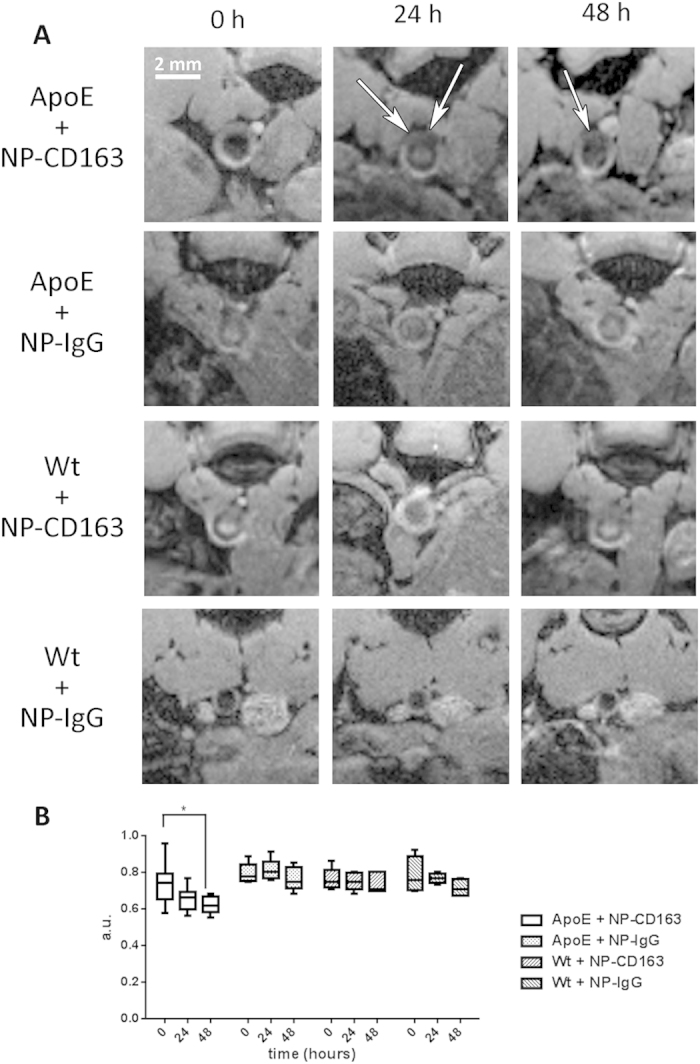
Plaque detection in apoE−/− mouse model. (**A**) Representative magnetic resonance images obtain before (0 h) and after (24 h, 48 h) the injection of each type of nanoparticles. The white arrows point at slightly darker areas with respect to the pre-injection images in the case of apoE−/− mice injected with NP-CD163(m). (**B**) Graph showing the normalized mean value of the abdominal aortic wall for apoE−/− and wild type mice injected with either NP-CD163(m) or NP-IgG(m) at 0, 24 and 48 hours. The error bars represent the SEM. *p < 0.05, **p < 0.01 and ***p < 0.001 *versus* apoE + NP-CD163 t = 48h.

ApoE−/− and wild type mice were both injected in separate experiments with either NP-CD163(m) or NP-IgG(m) and the pararenal abdominal aorta was imaged at four time points: before the injection and at 1, 24 and 48 hours post-injection. No differences were seen at 1hour post-injection (data not shown), but a signal decrease in the aortic wall was observed in apoE−/− mice injected with NP-CD163 (n = 9) over time with respect to the pre-injection signal. At 24 hours there was a signal decrease that became significant at 48 hours after the injection (p < 0.01). Conversely, when the same type of mice were injected with the control probe NP-IgG(m) there were not significant signal variations over time or in healthy mice (See Supplementary tables S7-S9 for statistical details). Likewise, the wall signal intensity did not vary compared to pre-injection signal in the case of wild type mice injected with either NP-CD163(m) or NP-IgG(m) (Fig. 3).

Finally, 4 of the apoE−/− mice that were injected with NP-CD163 were scanned in the MRI 6 days after the injection to study the clearance of the nanoparticles, and a recovery of the signal comparable to pre-injection signal intensity was observed (Figure S5 in supplemental material).

### Immunohistochemistry validation

**Figure 4:**
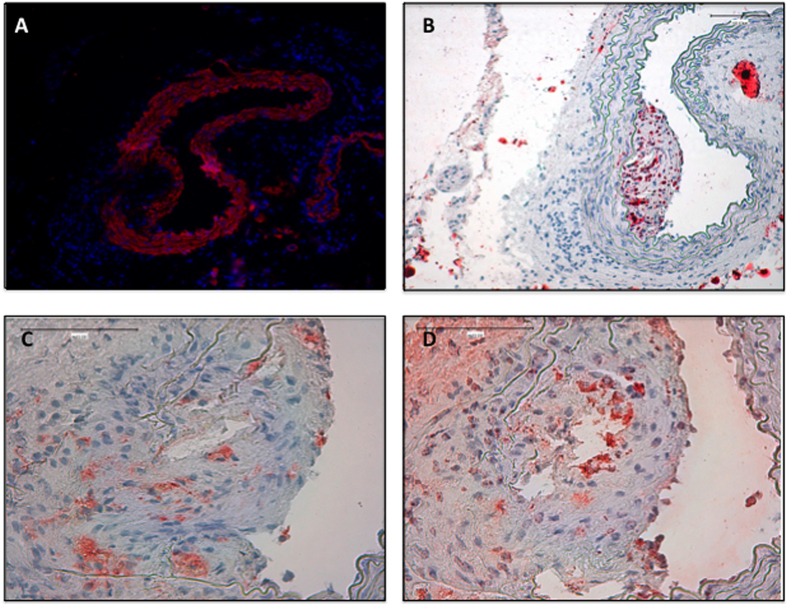
Validation of the plaque presence and molecular biomarkers in apoE−/− mice. Representative micrographs of aortic serial sections isolated from apoE−/− NP CD163(m) injected mice. (**A**) Vascular smooth muscle staining by α-actin immunohistochemistry detection, (**B**) Lipid determination by Oil red staining (**C**) CD68 immunohistochemistry detection and (**D**) CD163 immunohistochemistry detection. (n = 7).

The histology of the apoE−/− aortas, harvested after MRI scan, showed that all the mice had atherosclerotic lesions in the MRI scanned area (Fig. 4) with an average size of 50,000 ± 14,000 μm^2^. The plaques in the aortic wall were composed by a layer of smooth muscle cells (Fig. 4A) with small lipid deposits (oil red staining, Fig. 4B) and with the presence of infiltrated macrophages (CD68, Fig. 4C). MRI results were confirmed in the atherosclerotic mice samples by CD163 (Fig. 4D), and CD68 staining. The incubation with an isotype-matched immunoglobulin was used as a negative control, showing the specificity of the immunohistochemistry (Supplemental Figure S6).

## Discussion

Imaging macrophages in atherosclerosis is an appealing strategy since they play an important role in the development, growth and rupture of atherosclerotic plaque. In particular, the use of targeted probes specifically directed to the detection of subtypes of macrophages could provide useful information on the composition of the plaque. For these reasons, the use of a targeted probe detection of CD163-expressing macrophages in atherosclerosis by MRI could be of interest, because these types of macrophages, although not exclusively, are accumulated in hemorrhaged or asymptomatic plaques^5^,^7^.

Iron oxide nanoparticles are an alternative to gadolinium based contrast agents for patients with renal disorders due to nephrogenic systemic fibrosis that the gadolinium may induce^27^,^28^. Moreover, by coating those iron oxide nanoparticles with gold we achieved a very versatile platform due to the easy functionalization of the NP surface by using thiol-ending ligands taking advantage of the stability of gold-sulphur bond. Hence, following an established procedure we were able to successfully prepare and characterize gold-coated iron oxide nanoparticles linked to anti-CD163 antibodies. Given their high specificity and availability against many proteins or their modifications, antibodies have been successfully used as vectors for the targeting of many proteins, also in atherosclerosis^29^. The oriented grafting of IgG antibodies on the nanoparticles by the Fc leaving the Fab exposed for recognition was a challenge that was overcome by the use of protG. Such protein is known to bind to IgG antibodies in the desired oriented manner^23^. The amount of protG-antibody complex ranged from 1 to 2 units per nanoparticle, which is a desirable amount to ensure that a greater number of nanoparticles link to the receptors. This platform may also be able to link any other antibody binding proteins (A, L or recombinant A/G) allowing also the conjugation of non-IgG antibodies that would potentially enable us to vectorized the probe with any type of antibody.

Subsequently, the probe was tested *in vitro* by incubation of the targeted probe (NP-CD163) and the negative control probe (NP-IgG) with both CD163 (+) and (−) macrophages. The same trend was observed in both human and mice cells and a significant decrease of the T_2_ values were observed when NP-CD163 was incubated with macrophages expressing CD163, whereas the T_2_ value did not decrease in a significant manner when the same cells were incubated with the control probe NP-IgG. From these data we concluded that the probe specifically targeted macrophages that expressed CD163, but in order to prove that the specificity of the nanoparticle was due to CD163 receptor recognition we performed inhibition experiments incubating with increasing concentration of free antibody before probe targeting. The unlabelled antibody blocked the targeting of CD163 (+) macrophages with the NP-CD163 probe both in human and in mice cells. It is noteworthy that one equivalent of free antibody was able to block completely the NP-CD163 T_2_ signal. This can be explained because the NP-CD163 probe is more steric hindered than the free antibody and/or the link of the antibody to the nanoparticle might decrease the affinity for its target. Additional control experiments were performed to exclude a potential mannose-directed binding of the NPs to macrophages. For this purpose NPs without ProtG and antibodies were incubated with both CD163 (+) and (−) cells and the binding analyzed by MRI. Those NPs bound abundantly and indiscriminately to both types of cells due to the presence of mannose receptors (data not shown), however, when the NPs were functionalized with IgG antibodies we only detected significant binding when NP-CD163 was incubated with CD163 (+) cells (Fig. 2B,E). To sum up, all these results demonstrated that the probe was accumulated by CD163 (+) macrophages only through their interaction with the antibody. Hence the selected probe was suitable for the selective detection of M2-macrophages with respect to pro-inflammatory M1-macrophages in both human and mice cells.

Once the specificity of the probe was tested *in vitro*, the targeted and control probes were injected in mice developing atherosclerotic lesions and wild type mice as healthy controls. As shown by the images, the accumulation of probe in the site of interest increased over time and became significant 48 hours after the injection in atherosclerotic mice. The fact that the probe is not accumulated in healthy aorta reinforces the fact that the MRI signal is due to the specific labelling of the NP-CD163(m) probe (see supplemental Figures S7-S8). The probe is cleared in time in the aorta because its presence was not detected *via* MRI 6 days post-injection. Interestingly, none of the negative controls showed a decrease in the wall intensity signal over time. Indeed, apoE−/− mice injected with NP-IgG(m) and wild type mice injected with either NP-CD163(m) or NP-IgG(m) did not present any significant changes during 48 hours after injection with respect to the signal intensity of the aortic wall before the injection. Hence, the targeted probe designed by us seems to be able to selectively detect CD163 receptor in the aortic wall of mice presenting atherosclerotic lesions. Finally, the *in vivo* results obtained by MRI were confirmed using immunohistochemistry techniques showing the presence of atherosclerotic plaques with CD163(+) macrophages in the lesions of the mice.

In conclusion, we have proved the versatility of our construction by linking it with different antibodies. The FA-11 and M-96 antibodies linked in our construction were capable to detect CD163 *in vitro* and the M-96 antibody, recognizing the mouse CD163 receptor, linked to our NP was capable of detecting a subpopulation of macrophages in atheromatous plaques *in vivo*.

## Methods

### Antibodies

Monoclonal anti-human CD163 (AbDserotec, MCA1853), mouse IgG1 negative control (AbDserotec, MCA928), rabbit polyclonal anti-mouse CD163 (M-96, Santa Cruz, sc-33560), rabbit IgG negative control (Santa Cruz, sc-2027), monoclonal Anti-α Smooth Muscle Actin (Sigma-Aldrich, A-2547), rat monoclonal anti-mouse CD68 (FA-11, Abcam, ab53444) were used for MRI, western-blot and immunohistochemistry detection.

### Cell lines and culture medium

The human monocytic cell line THP-1 [American Type Culture Collection (ATCC), Rockville, MD] and murine peritoneal macrophages, isolated as previously described^30^, were cultured in RPMI (BioWittaker) supplemented with 10% FBS (BioWittaker), 2 mM L-glutamine, 100 U/ml penicillin and 100 μg/ml streptomicin (Invitrogen). THPs were differentiated to macrophages using PMA (10^−7^ M) for 48 h. After that, THP-1 macrophages cells and murine peritoneal macrophages were treated for 24 h with dexamethasone (2.5 × 10^−7 ^M) to induce CD163 expression.

### Synthesis of gold-coated iron oxide nanoparticles, functionalization with IgG antibodies and characterization

Hexane soluble oleic acid protected iron oxide seeds (Fe_3_O_4_@oleic NPs) and gold coated iron oxide nanoparticles (Fe_3_O_4_@Au@oleic NPs) herein reported were prepared as described before following a modification of a procedure reported by Wang *et al.*^22^,^31^ (Supplemental Figure S1 and S2). These nanoparticles were characterized by TEM on a JEOL JEM 2100F microscope before and after the gold coating and their composition was determined by XPS analysis on a SPECS Sage HR 100 spectrometer, also before and after the coating (Supplemental Figure S3). As previously reported, oleic acid protected gold-coated iron oxide nanoparticles (Fe_3_O_4_@Au@oleic NPs) underwent a ligand exchange step with a 1:1 mixture of carboxylic acid ending ligands and mannose ending ligands to yield water soluble Fe_3_O_4_@Au nanoparticles (Fe_3_O_4_@Au@Man/CO_2_H NPs). These nanoparticles were characterized by TEM, UV-Vis and ICP-OES for the quantification of the gold and iron content. Subsequently, water soluble Fe_3_O_4_@Au@Man/CO_2_H NPs were converted into targeted NPs through a peptide coupling with amine groups present in protein G (protG, Pierce^®^Ig Binding protein, Thermo Scientific) and further incubation of IgG antibodies with the NP-protG complex, following a two step procedure described by us for the same nanoparticles^23^. Both human and mouse anti-CD163 and their corresponding IgG isotype antibodies were conjugated to the NP-protG complex leading to the formation of 4 probes: NP-CD163(h) and NP-IgG(h) for human antibodies, and NP-CD163(m) and NP-IgG(m) for mouse antibodies. The amount of protG and antibody on the nanoparticles was separately determined by Bradford assay of the unbound protG and the antibody recovered in the washings after the peptide coupling and the antibody incubation, respectively, as described before^23^. Additionally, the presence of the antibodies was confirmed by SDS-PAGE gels (Supplemental Figure S4).

### Specificity studies of nanoparticles

*In vitro* targeting was pursued to demonstrate the specificity of NP-CD163. In order to accomplish that, two cell lines were used, human THP-1 monocyte-macrophage cell line and isolated murine macrophages. Macrophages are able to overexpress CD163 under stimulation of dexamethasone as described in Schaer *et al.*^32^ and its expression was validated by Western-blot or immunofluorescence (Fig. 2A). 10^6^ human cells, expressing or not CD163, were incubated with either NP-CD163(h) or NP-IgG(h) (as antibody control) at 4 °C, employing 1.5 μg of Fe per 10^6 ^cells in 300 μL of PBS. After 1 hour the cells were centrifuged to remove the unbound NPs and several washings with PBS were performed. The incubated cells were placed in capillary tubes and allowed to form a pellet overnight at 4 °C. Those capillary tubes were inserted in agarose gel (2% w/w) to prepare phantoms for MRI. The same incubation protocol was applied to murine cells that expressed or not CD163 but using 5×10^5 ^cells per incubation. Inhibition studies with both cell lines were performed with increased amounts of free antibody (1:1 and 1:10 with respect to the probe), in order to block the NP-CD163 binding to ensure that the T_2_-signal obtained was due to the specificity of the antibody. All incubation and inhibition experiments were performed at least 3 times.

### Animal model

8 weeks old B6.129P2-apoE^tm1Unc^/J transgenic mice (apoE−/−, n = 5–9) as atherosclerotic model and healthy wild type mice as control (Wt, n = 3–5) (Jackson Laboratory, Bar Harbor, ME) were used for *in vivo* studies. apoE−/− mice were fed during 8 weeks with high-fat diet (TD.88137 42% Fat, Harlan Teklan, Madison, Wisconsin) to induce atherosclerosis development. Mice were anesthetized for all procedures (isoflurane 2% to 3% v/v, Baxter). The NP-CD163(m) and its negative control NP-IgG(m) were injected in a dose of 2 mg Fe/Kg mice intravenously. The institutional subcommittee (Provincial Council of Guipuzcoa) on research animal care approved all animal studies, all the studies were carried out in accordance with the approved guidelines.

### MR Imaging

Measurements were made on an 11.7 Tesla horizontal bore BrukerBiospec 117/16 scanner (Bruker A.G., Ettlingen, Germany). The actively shielded BFG-150/90-S gradient insert (Resonance Research Inc. Ballerica, MA, USA) has a 90 mm gradient coils are capable of switching 750 mT/m within 100 μs.

### Imaging of cells phantoms

T_2_ relaxation times were calculated from images obtained using a multiple spin echo sequence with equally spaced 64 echoes ranging from 10 ms to 640 ms and 12000 ms repetition time. The in plane resolution was 133 × 133 μm^2^ and the slice thickness varied between 300 μm and 500 μm in the different samples. The slice thickness was varied to avoid partial volume effects from the water above the pellet of cells, as the volume of pellet could vary from sample to sample. Each echo was acquired 8 times to improve the signal to noise ratio.

### Imaging of mice

*In vivo* images were acquired using a respiratory-triggered flow compensated gradient recalled echo sequence. The sequence parameters were the following: TR = ~1 breathing; TE = 4 ms; FA = 30°; number of averages = 1; FOV = 2 × 2 cm; in plane resolution = 78 × 78 μm, slice thickness = 0.5 mm. Four adjacent slabs, each containing five slices, of the pararenal abdominal aorta were acquired in each experiment with a 1 mm saturation band at 2.5 mm to suppress the downstream flow entering the imaged slices. Images of the same mice were acquired before injection and 1, 24 and 48 hours after injection.

### Histopathology and immunohistochemistry

To validate the *in vivo* MRI imaging, equivalent sections of the scanned aortas were harvested and embedded in OCT. Serial 4-μm–thick cryostat sections were stained either with oil red for lipid deposits detection and characterization with the following antibodies: CD163 and CD68 (macrophages), and α-actin (smooth muscle cells). The secondary antibodies and ABComplex/HRP were added and sections were stained with 3, 30-diaminobenzidine, counterstained with hematoxylin and mounted in DPX. For CD163 detection Tyramide Signal Amplification Biotin Kit (PerkinElmer, NEL700A001KT) was needed to improve signal detection. Images were taken using a Nikon Eclipse E400 microscope and Nikon ACT-1 software.

### Image analysis

MRI and histology images were analyzed using Image J (Image J 1.47v, NIH, USA). Signal intensity of the cell pellets placed inside capillary tubes was quantified in each slice for each echo time by drawing ROIs (region of interest) on them. Subsequently a monoexponential decay curve was fitted to the obtained results to calculate T2 values in each capillary tube using Origin (OriginLab, Northampton, MA). The T2 values calculated for each cell pellet were normalized to the T2 value of the blank sample (cells without NPs) and compared among them to determine the selectivity of the probes. For the *in vivo* experiments, the aortic vessel wall and the adjacent spinal muscle were manually segmented to determine the signal-to-noise-ratio (SNR) of each. The SNR of the aortic wall was normalized to the SNR of the adjacent muscle on each slice and averaged for each animal and time point. Plaque size was quantified in two slices of two sections separated from 100 μm and next to the pararenal aorta.

### Statistics

Results are expressed as mean ± SEM. The data among groups were compared by using either 1-way ANOVA followed by DMS´s or Games Howell´s post hoc test for multiple comparisons depending on Levene’s test for homogeneity of variances. It was considered statistically significant when p-value ≤ 0.05.

## Additional Information

**How to cite this article**: Tarin, C. *et al.* Targeted gold-coated iron oxide nanoparticles for CD163 detection in atherosclerosis by MRI. *Sci. Rep.* **5**, 17135; doi: 10.1038/srep17135 (2015).

## Supplementary Material

Supplementary Information

## References

[b1] Fuster V. *et al.* Atherothrombosis and high-risk plaque-Part II: Approaches by noninvasive computed tomographic/magnetic resonance imaging. J. Am. Coll. Cardiol. 46, 1209–1218 (2005). 16198833 10.1016/j.jacc.2005.03.075

[b2] Go A. S. *et al.* Executive Summary: Heart Disease and Stroke Statistics-2013 Update A Report From the American Heart Association. Circulation 127, 143–152 (2013). 23283859 10.1161/CIR.0b013e318282ab8f

[b3] Etzerodt A. & Moestrup S. K. CD163 and Inflammation: Biological, Diagnostic, and Therapeutic Aspects. Antioxid. Redox Signal. 18, 2352–2363 (2013). 22900885 10.1089/ars.2012.4834 PMC3638564

[b4] Ley K., Miller Y. I. & Hedrick C. C. Monocyte and Macrophage Dynamics During Atherogenesis. Arterioscler. Thromb. Vasc. Biol. 31, 1506–1516 (2011). 21677293 10.1161/ATVBAHA.110.221127 PMC3133596

[b5] Stoger J. L. *et al.* Distribution of macrophage polarization markers in human atherosclerosis. Atherosclerosis 225, 461–468 (2012). 23078881 10.1016/j.atherosclerosis.2012.09.013

[b6] Finn A. V. *et al.* Hemoglobin Directs Macrophage Differentiation and Prevents Foam Cell Formation in Human Atherosclerotic Plaques. J. Am. Coll. Cardiol. 59, 166–177 (2012). 22154776 10.1016/j.jacc.2011.10.852 PMC3253238

[b7] Cho K. Y. *et al.* The Phenotype of Infiltrating Macrophages Influences Arteriosclerotic Plaque Vulnerability in the Carotid Artery. J. Stroke Cerebrovasc. Dis. 22, 910–918 (2013). 23273713 10.1016/j.jstrokecerebrovasdis.2012.11.020

[b8] Zorzi P., Aplin A. C., Smith K. D. & Nicosia R. F. The rat aorta contains resident mononuclear phagocytes with proliferative capacity and proangiogenic properties. J. Leukocyte Biol. 88, 1051–1059 (2010). 20628067 10.1189/jlb.0310178 PMC3072232

[b9] Hasan D., Chalouhi N., Jabbour P. & Hashimoto T. Macrophage imbalance (M1 vs. M2) and upregulation of mast cells in wall of ruptured human cerebral aneurysms: preliminary results. J. Neuroinflammation 9 222 (2012). 22999528 10.1186/1742-2094-9-222 PMC3488554

[b10] Sadeghi M. M., Glover D. K., Lanza G. M., Fayad Z. A. & Johnson L. L. Imaging Atherosclerosis and Vulnerable Plaque. J. Nucl. Med. 51, 51S–65S (2010). 20395341 10.2967/jnumed.109.068163 PMC2911776

[b11] Kanwar R. K., Chaudhary R., Tsuzuki T. & Kanwar J. Emerging engineered magnetic nanoparticulate probes for targeted MRI of atherosclerotic plaque macrophages. Nanomedicine 7, 735–749 (2012). 22630154 10.2217/nnm.12.46

[b12] Ruehm S. G., Corot C., Vogt P., Kolb S. & Debatin J. F. Magnetic resonance imaging of atherosclerotic plaque with ultrasmall superparamagnetic particles of iron oxide in hyperlipidemic rabbits. Circulation 103, 415–422 (2001). 11157694 10.1161/01.cir.103.3.415

[b13] Weissleder R. *et al.* Ultrasmall Superparamagnetic Iron-Oxide-Characterization of a New Class of Contrast Agents for MR Imaging. Radiology 175, 489–493 (1990). 2326474 10.1148/radiology.175.2.2326474

[b14] Kooi M. E. *et al.* Accumulation of ultrasmall superparamagnetic particles of iron oxide in human atherosclerotic plaques can be detected by *in vivo* magnetic resonance imaging. Circulation 107, 2453–2458 (2003). 12719280 10.1161/01.CIR.0000068315.98705.CC

[b15] El-Dakdouki M. H. *et al.* CD44 Targeting Magnetic Glyconanoparticles for Atherosclerotic Plaque Imaging. Pharm. Res. 31, 1426–1437 (2013). 23568520 10.1007/s11095-013-1021-8 PMC3823634

[b16] Amirbekian V. *et al.* Detecting and assessing macrophages *in vivo* to evaluate atherosclerosis noninvasively using molecular MRI. Proc. Natl. Acad. Sci. USA 104, 961–966 (2007). 17215360 10.1073/pnas.0606281104 PMC1766334

[b17] Lipinski M. J. *et al.* Macrophage-Specific Lipid-Based Nanoparticles Improve Cardiac Magnetic Resonance Detection and Characterization of Human Atherosclerosis. JACC Cardiovasc. Imaging 2, 637–647 (2009). 19442953 10.1016/j.jcmg.2008.08.009 PMC2756539

[b18] Yamakoshi Y. *et al.* LDL-based nanoparticles for contrast enhanced MRI of atheroplaques in mouse models. Chem. Commun. 47, 8835–8837 (2011). 10.1039/c1cc10924c 21743892

[b19] Li D. *et al.* Molecular Imaging of Atherosclerotic Plaques Targeted to Oxidized LDL Receptor LOX-1 by SPECT/CT and Magnetic Resonance. Circ. Cardiovasc. Imaging 3, 464–472 (2010). 20442371 10.1161/CIRCIMAGING.109.896654 PMC2955298

[b20] Briley-Saebo K. C. *et al.* Targeted molecular probes for imaging atherosclerotic lesions with magnetic resonance using antibodies that recognize oxidation-specific epitopes. Circulation 117, 3206–3215 (2008). 18541740 10.1161/CIRCULATIONAHA.107.757120 PMC4492476

[b21] Briley-Saebo K. C. *et al.* Targeted Iron Oxide Particles for *In Vivo* Magnetic Resonance Detection of Atherosclerotic Lesions With Antibodies Directed to Oxidation-Specific Epitopes. J. Am. Coll. Cardiol. 57, 337–347 (2011). 21106318 10.1016/j.jacc.2010.09.023 PMC3095034

[b22] Gallo J., Garcia I., Padro D., Arnaiz B. & Penades S. Water-soluble magnetic glyconanoparticles based on metal-doped ferrites coated with gold: Synthesis and characterization. J. Mater. Chem. 20, 10010–10020 (2010).

[b23] Garcia I., Gallo J., Genicio N., Padro D. & Penades S. Magnetic Glyconanoparticles as a Versatile Platform for Selective Immunolabeling and Imaging of Cells. Bioconjugate Chem. 22, 264–273 (2011). 10.1021/bc1003923 21247095

[b24] Gallo J., Garcia I., Genicio N., Padro D. & Penades S. Specific labelling of cell populations in blood with targeted immuno-fluorescent/magnetic glyconanoparticles. Biomaterials 32, 9818–9825 (2011). 21940045 10.1016/j.biomaterials.2011.09.010

[b25] Elvira G. *et al.* Live Imaging of Mouse Endogenous Neural Progenitors Migrating in Response to an Induced Tumor. PloS One 7, e44466 (2012). 22957072 10.1371/journal.pone.0044466 PMC3434138

[b26] Carril M., Fernandez I., Rodriguez J., Garcia I. & Penades S. Gold-Coated Iron Oxide Glyconanoparticles for MRI, CT, and US Multimodal Imaging. Part. Part. Syst. Charact. 31, 81–87 (2014).

[b27] Canavese C. *et al.* Gadolinium-associated nephrogenic systemic fibrosis: the need for nephrologists’ awareness. J. Nephrol. 21, 324–336 (2008). 18587720

[b28] Kay J. Nephrogenic systemic fibrosis: a gadolinium-associated fibrosing disorder in patients with renal dysfunction. Ann. Rheum. Dis. 67 Suppl 3, iii66-69 (2008). 10.1136/ard.2008.102475 19022818

[b29] Boekhorst B. C. M. T., Cramer M.-J. M., Pasterkamp G., van Echteld C. J. A. & Doevendans P. A. F. M. Recent developments and new perspectives on imaging of atherosclerotic plaque: role of anatomical, cellular and molecular MRI part III. Int. J. Cardiovasc. Imaging 26, 447–457 (2010). 20058083 10.1007/s10554-009-9566-7

[b30] Ray A. & Dittel B. N. Isolation of mouse peritoneal cavity cells. J. Vis. Exp. 35, e1488 (2010). 10.3791/1488 PMC3152216 20110936

[b31] Wang L. Y. *et al.* Monodispersed core-shell Fe3O4@Au nanoparticles. J. Phys. Chem. B 109, 21593–21601 (2005). 16853803 10.1021/jp0543429

[b32] Schaer D. J., Boretti F. S., Schoedon G. & Schaffner A. Induction of the CD163-dependent haemoglobin uptake by macrophages as a novel anti-inflammatory action of glucocorticoids. Br. J. Haematol. 119, 239–243 (2002). 12358930 10.1046/j.1365-2141.2002.03790.x

