# Supplementary material for: Targeted gold-coated iron oxide nanoparticles for CD163 detection in atherosclerosis by MRI
Source: Sci Rep. 2015 Nov 30;5:17135. doi: 10.1038/srep17135 (PMC4663748; doi:10.1038/srep17135)
Supplement: Supplementary Information [file srep17135-s1.doc]

**Targeted gold-coated iron oxide nanoparticles for CD163 detection in atherosclerosis by MRI.**

Carlos Tarin, Monica Carril, Jose Luis Martin-Ventura, Irati Markuerkiaga, Daniel Padro, Patricia Llamas-Granda, Juan Antonio Moreno, Isabel García, Nuria Genicio, Sandra Plaza-Garcia, Luis Miguel Blanco-Colio, Soledad Penades, Jesus Egido

**SUPPLEMENTAL MATERIAL**

**1. Synthesis of ligands.**

Mannose ligand **4** was prepared through a glycosidation reaction between the per-acetilated mannose **1** and linker **2** (purchased from Aldrich) followed by a deprotection step with sodium methoxide (Scheme S1A). The carboxylic ligand **6** is obtained through a Jones oxidation of linker **2** followed by deprotection with sodium methoxide (Scheme S1B). Both mannose and carboxylic ligands are obtained as a 1:1 mixture of thiol and disulfide derivative. which were used in the probe preparation without further purification. For detailed experimental procedures see García *et al.* [1].


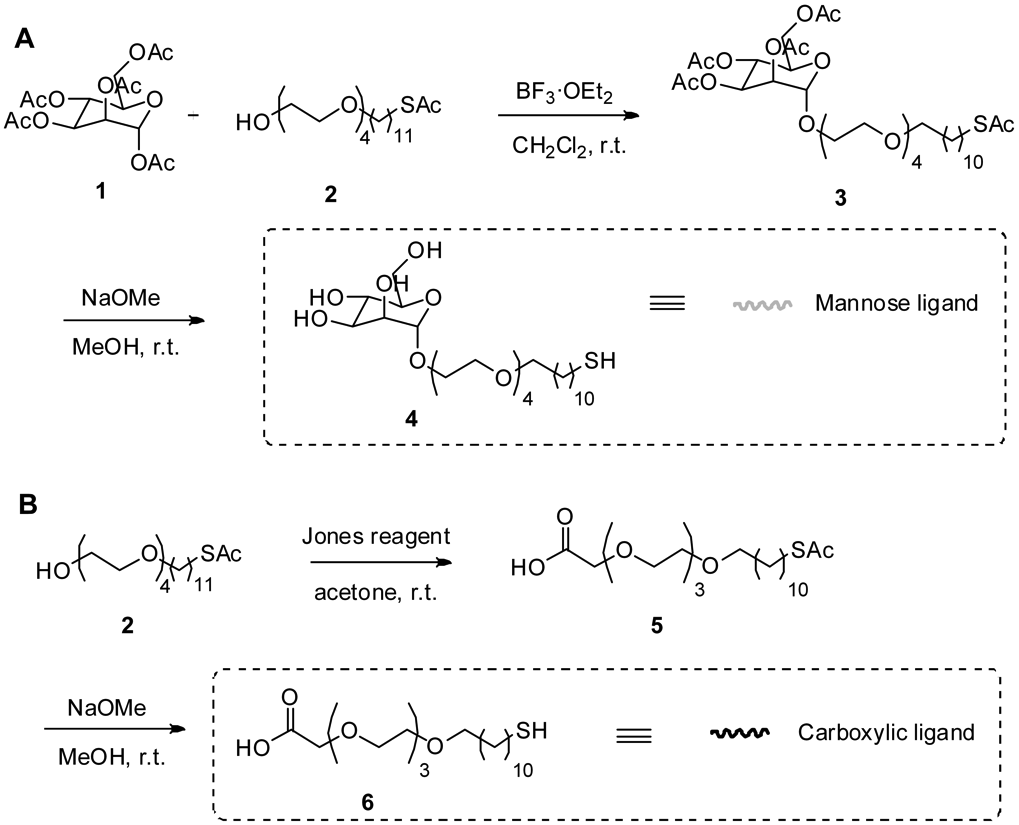


**Scheme S1.** (A) Mannose ligand synthesis. (B) Carboxylic ligand synthesis.

**2. Synthesis of probe.**

The probe was prepared in a stepwise process which began with the magnetic core synthesis and its gold coating process (Scheme S2A). Once the metallic core was prepared. a ligand exchange was performed employing amphiphilic mannose and carboxylic ligands to render the nanoparticles water soluble (Scheme S2B). Afterwards. protG was linked to the carboxylic ending ligands through a peptide bond. followed by the conjugation of the selected IgG antibody (Scheme S2C). For detailed procedures for the metallic core preparation and ligand exchange see Gallo *et al.* [2] and for the protG-IgG conjugation see García *et al.* [1].


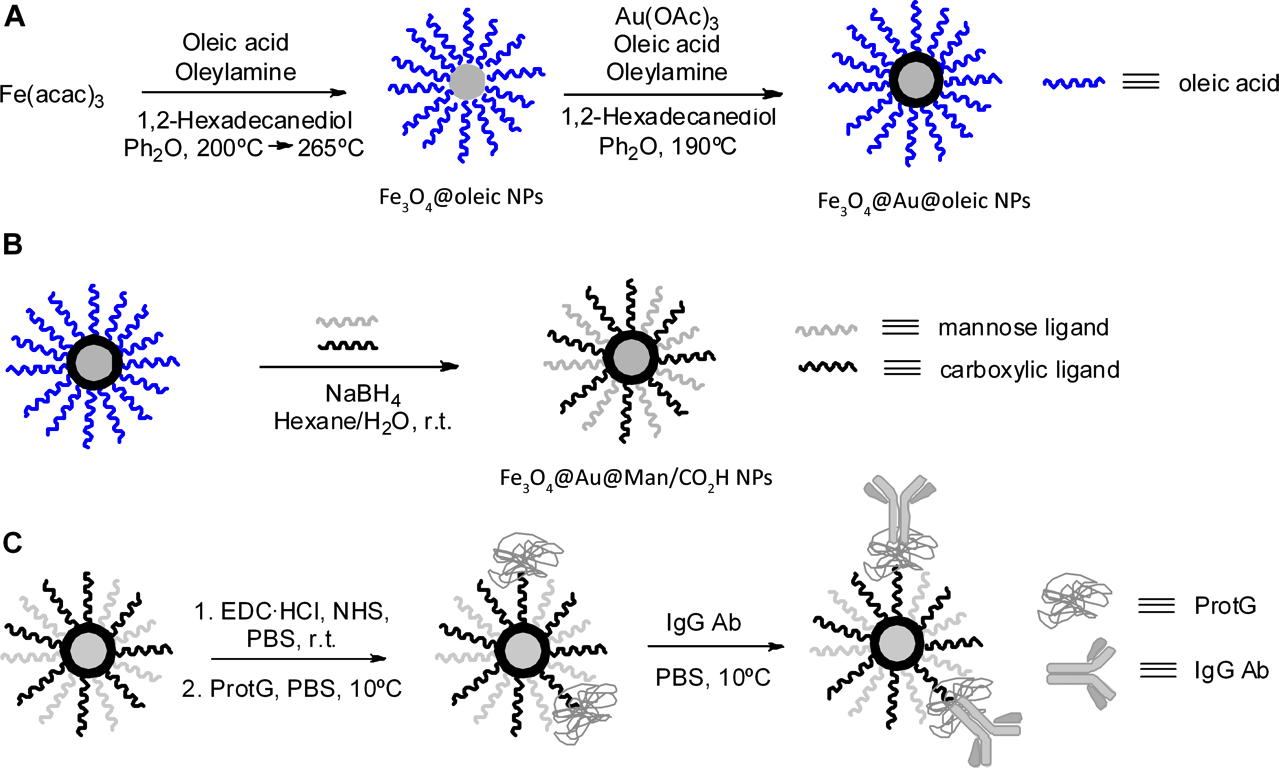


**Scheme S2**. (A) oleic acid protected metallic core synthesis. (B) Ligand exchange process to produce water soluble Fe_3_O_4_@Au@Man/CO_2_H NPs. (C) ProtG and IgG antibody conjugation steps.

**3. XPS measurements**.

XPS experiments were performed in a SPECS Sage HR 100 spectrometer with a non‑monochromatic X‑ray source (Magnesium K_α_ line of 1253.6 eV energy and 250 W) and calibrated using the 3d_5/2_ line of Ag with a full width at half maximum (FWHM) of 1.1 eV. The selected resolution for the spectra was 15 eV of Pass Energy and 0.15 eV/step. All Measurements were made in an ultra high vacuum (UHV) chamber at a pressure below 5·10^‑8^ mbar. An electron flood gun was used to compensate for charging during XPS data acquisition. In the fittings asymmetric functions were used (after a Shirley background correction) where the FWHM of the peaks were constrained while the peak positions and areas were set free. The samples measured were F_3_O_4_@oleic NP and F_3_O_4_@Au@oleic NP.

*High resolution spectra of Fe 2p peak*

Figure S3A shows the Fe 2p spectra of the samples showing the expected profile for Fe_3_O_4_.[3. 4]

*High resolution spectra of Fe 3p peak*

Figure S3B shows the Fe 3p spectra of both samples. The normalized Fe 3p spectra (data not shown) show that they have the same shape which means that the iron oxide core remained unchanged after the gold coating process.

*Fitting of the Fe 3p_3/2_ peak*

Figure S3C and D show the fitted Fe 3p spectra of both samples. The calculated Fe^3+^/Fe^2+^ ratio for both samples is approximately 2 which is the theoretical expected ratio for Fe_3_O_4_.

*High resolution spectrum of Au 4f peak*

Figure S3E shows the Au 4f spectrum of the Fe_3_O_4_@Au@oleic NP sample. The Au 4f_7/2_ peak in the sample shows that the Au is Au(0). [3]


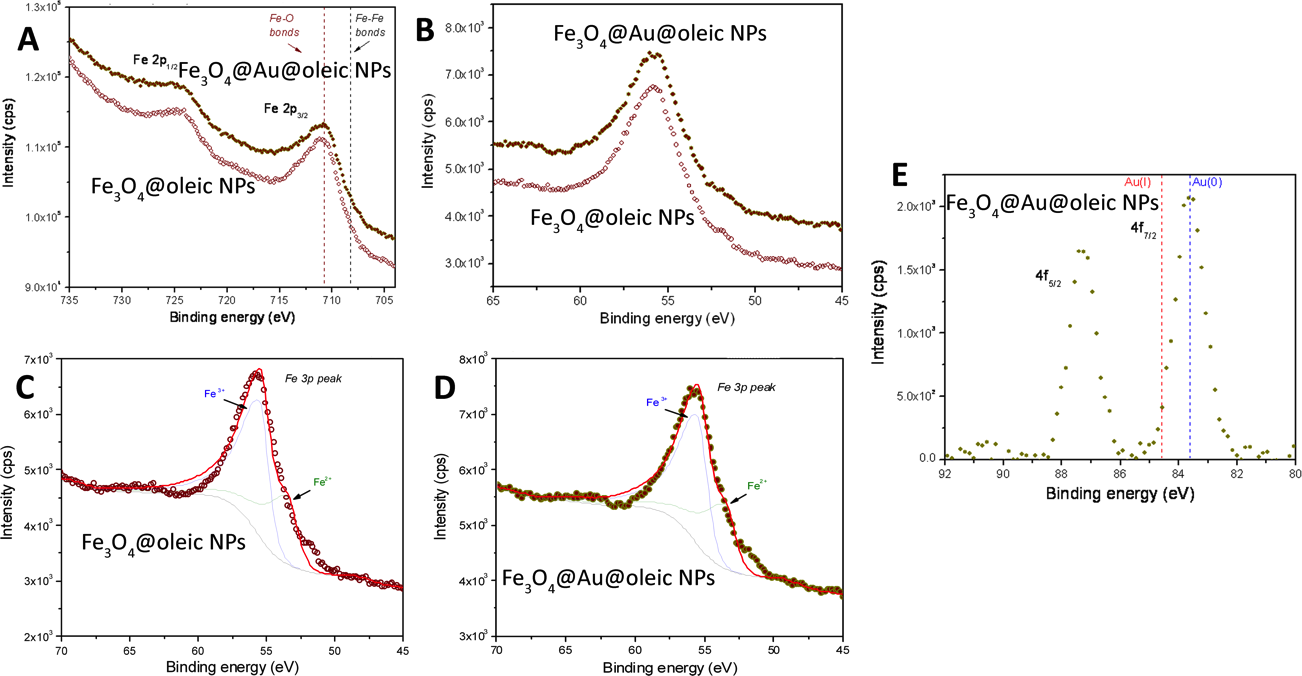


**Figure S3.** (A) High resolution spectra of Fe 2p peak of F_3_O_4_@oleic NP and F_3_O_4_@Au@oleic NP. (B) High resolution spectra of Fe 3p peak of F_3_O_4_@oleic NP and F_3_O_4_@Au@oleic NP. (C) Fitting of the Fe 3p_3/2_ peak of F_3_O_4_@oleic NP. (D) Fitting of the Fe 3p_3/2_ peak of F_3_O_4_@Au@oleic NP. (E) High resolution spectrum of Au 4f peak of F_3_O_4_@Au@oleic NP.

**4. SDS-Page gel.**

An acrylamide gel was prepared following a Laemmly standard protocol. The cross linking employed was 4% and 12% for the stacking and resolving gels, respectively. For sample preparation each 20 µL aliquot of free antibody, nanoparticles bearing antibodies, and molecular weight marker was heated at 95°C for 5min with 10 µL of sample buffer (62.5 mM Tris-HCl pH= 6.8. 20% glycerol. 2% SDS. 5% B-mercaptoethanol). Samples were seeded on the gel and then ran using SDS running buffer (25 mM Tris, 192 mM glycine, 0.1% SDS, pH = 8.3) for 45 minutes at 100 V and 15 minutes at 250 V. Finally, Coomassie staining protocol was applied to reveal the bands. All four IgG antibodies loaded onto the nanoparticles showed the same behaviour in the SDS-Page and hence in Figure S4 mouse anti-CD163 antibody is shown as a representative example. IgG antibodies showed two bands at 25KDa and 50KDa after digestion which were also visible in the rows where the antibody bearing nanoparticles were seeded.[1]


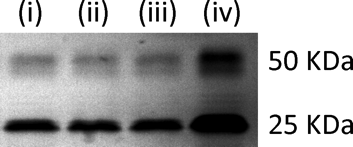


**Figure S4**. (i) NP-CD163 (h). (ii) NP-IgG(m). (iii) NP-CD163(m). (iv) mouse anti-CD163 antibody.

**5. Cell viability assays.**

In order to determine the toxicity of NP-CD163(m) in cell cultures, apoptosis (Anexin V) and viability assays (MTT) were performed (Figure S5) in RAW263.7.

Anexin V assay was performed using PE Anexin V Apoptosis Detection Kit I (559763, BD Pharmigen) according to manufacturer instructions in RAW264.7 cells. Anexin V staining was assessed using a FACSCantoII Citometer. As it is shown in the Figure S5A, Q4 population (Anexin V positive cells) are the apoptotic cells. There are no significant changes neither at 24 nor at 48 hours of exposure to NP-CD163(m) (Figure S5B) (ANOVA with a DMS post-hoc test).

MTT assay was performed in order to determine cell viability. 2.5·10^4^ RAW264.7 cells were seeded in p96 wells. Cells were depleted with culture medium at 0.5% of serum and incubated for 24 hours with dexamethasone to induce CD163 expression. CD163(+) RAW cells were then exposed to increasing amounts of NP-CD163(m) (range from 0 to 1000ng/mL) for 24 or 48 hours. After this time, cells were incubated in a solution of 0.5mg/mL Thiazolyl Blue Tetrazolium Blue (Sigma M5655) for 1 hour, the solution was removed and after the plate was dry the blue precipitate was resuspended with DMSO. Then, the absorbance was measured at 570nm with a microplate reader. The viability was referred to the control cells without NP. There is a significant but transient diminishing of cell viability in the RAW cells treated with the higher amount of NP compared to control group at 24 hours but it is recovered at 48 hours.


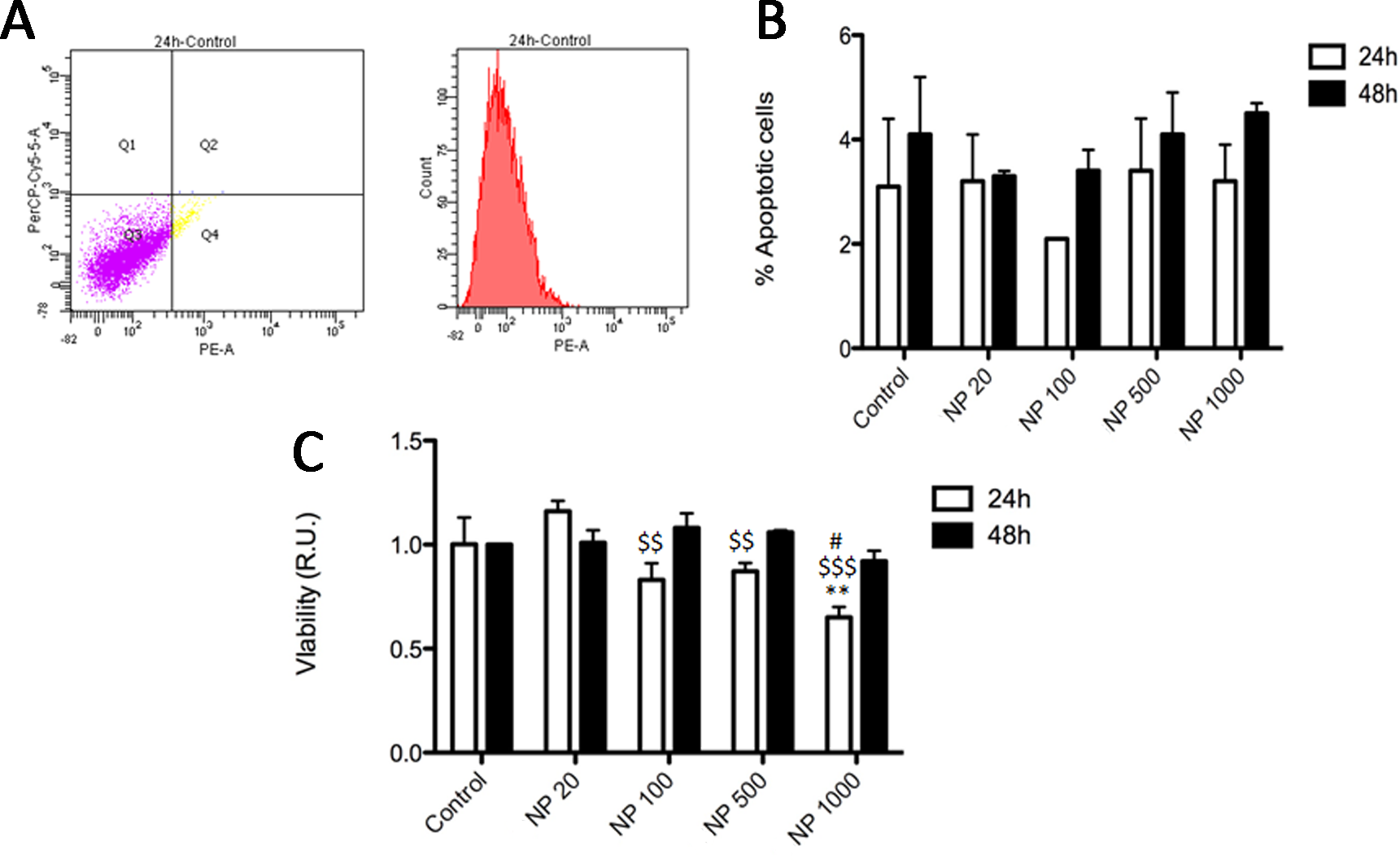


**Figure S5.** (A) Representative cytometry profile of Anexyn V staining in RAW 264.7. (B) Percentage of apoptotic cells (Q4) of RAW264.7 exposed to an increasing dose of NP-CD163(m) (0, 20, 100, 500 and 1000ng NP/mL named in the graph as Control, NP 20, NP 100, NP 500 and NP 1000, respectively). (C) Viability of RAW264.7 exposed to an increasing dose of NP-CD163(m) (0, 20, 100, 500 and 1000ng NP/mL named in the graph as Control, NP 20, NP 100, NP 500 and NP 1000, respectively). (ANOVA with a DMS post-hoc test) The error bars represent the SEM. ** p<0.01 versus Control, $$ p<0.01 and $$$ p<0.001 versus NP20 and # p<0.05 versus NP500.

**6. Toxicological profile, excretion and biodistribution.**

For the biodistribution and toxicity studies healthy 12 weeks-old apoE male mice were used. The animals were randomly distributed in 3 groups: 6 apoE mice were administered with 2 mg Fe/Kg of NP-CD163(m) (3 were euthanized at 24 hours post-injection and the other 3 at 48 hours post-injection) and 3 mice were used as controls injected with saline. Urine samples were collected at 24 and 48 hours after injection. Then, mice were anaesthetized and whole blood was extracted by cardiac puncture and collected in tubes with clot activator and gel for serum separation. Main organs (liver, spleen, kidney, heart and lung) and aorta were collected, weighted and fixed in formalin. All formalin fixed samples were paraffin embedded. Paraffin blocks containing spleen, liver, kidney, heart, aorta and lung were cut (4 μm) and Haemotoxylin-Eosin (HE)-stained, for histological evaluation (Figure S6).


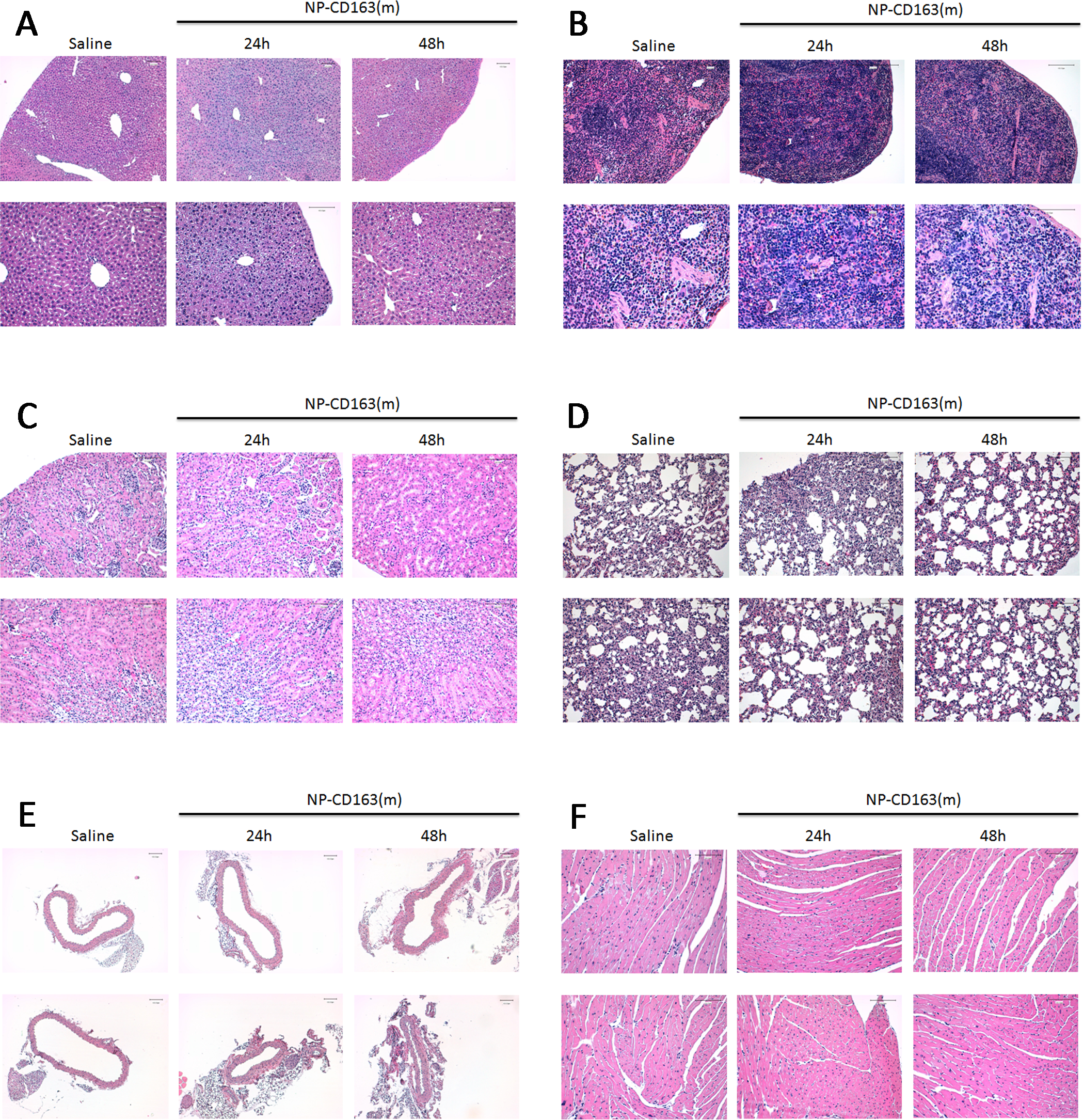


**Figure S6**. Hematoxylin-Eosin staining of tissue sections of different organs obtained from control mice injected with saline and mice 24 and 48 hours after being injected with NP-CD163 (m). (A) Tissue sections from liver. (B) Tissue sections from spleen. (C) Tissue sections from kidney. (D) Tissue sections from lung. (E) Tissue sections from aorta. (F) Tissue sections from heart.

Blood samples were centrifuged (10 min, 2000 rpm) and serum was frozen until biochemical evaluation. In total 10 parameters were evaluated: glucose (Glc), urea, blood urea nitrogen (BUN), total creatinin, total protein, albumin, calcium, aspartate transaminase (AST), alanine transaminase (ALT), alkaline phosphatase and total bilirubin content (Table S1).

| **Group** | **Control** | **NP-CD163(m) 24h** | **NP-CD163(m) 48h** |
| --- | --- | --- | --- |
| Weight (g) | 24,43±1,90 | 24,70±1,61 | 22,47±0,80 |
| Liver/weight | 0,046±0,008 | 0,050±0,008 | 0,048±0,004 |
| Kidney/weight | 0,012±0,001 | 0,013±0,001 | 0,012±0,001 |
| Spleen/weight | 0,012±0,001 | 0,013±0,001 | 0,012±0,001 |
| Lung/weight | 0,003±0,001 | 0,004±0,001 | 0,003±0,001 |
| Glc (mg/dl) | 114,7±19,2 | 186,7±21,8 | 159,7±37,3 |
| Urea (mg/dl) | 83,3±4,5 | 58,3±1,5*# | 73,3±1,7 |
| BUN (mg/dl) | 39,0±2,1 | 27,3±0,9*# | 34,3±0,7 |
| Total Creatinin (mg/dl) | 0,23±0,09 | 0,20±0,01 | 0,17±0,03 |
| Total Protein (g/dl) | 5,80±0,26 | 5,13±0,20 | 5,63±0,07 |
| Albumin (g/dl) | 3,37±0,12 | 3,00±0,17 | 3,17±0,03 |
| Calcium (mg/dl) | 9,00±0,32 | 8,80±0,31 | 9,03±0,12 |
| AST (UI/l) | 172,0±43,1 | 282,7±144,2 | 99,7±11,8 |
| ALT (UI/l) | 67,3±13,4 | 41,3±4,7 | 40,7±0,7 |
| Alkaline phosphatase (UI/l) | 101,7±6,3 | 134,0±8,7*# | 94,7±6,8 |
| Total Bilirubin (mg/dl) | 0,07±0,03 | 0,10±0,06 | 0,03±0,03 |

**Table S1.** Macroscopical characteristics and comparative biochemical profile between control group and injected group at 24 hours and 48 hours post-injection with NP-CD163(m) probe. * p<0.05 vs Control and # p<0.05 vs NP 48h (ANOVA with a DMS post-hoc test). N = 3 for each animal group.

Gold content was determined in some of the frozen samples (liver, spleen, kidney, lung and aorta) by inductively coupled plasma optical emission spectroscopy (ICP-OES) (Figure S7). Tissues were digested with aqua regia prior to their analysis.

| A | B |
| --- | --- |

**Figure S7**. (A) Gold content in ppm measured by ICP-OES for the main organs in apoE mice 48 hours after injection with NP-CD163(m). (B) Same results as in (A) expressed as percentage of injected dose. n. d. = not detected.

Gold staining allowed the detection of gold deposits in tissue sections from different organs obtained from mice injected with NP-CD163(m) 24 and 48 hours after administration. The gold staining was performed using the Silver Enhanced Kit (SE100, Sigma-Aldrich) according to manufacturer instructions and followed by a Prussian blue staining in order to differentiate the gold (black stain) of the iron deposits (blue). The observations were in agreement with the data obtained by ICP-OES analysis. As expected, gold was not detected in control mice injected with saline (Figure S8). As liver was the organ with highest accumulation of probe, an additional tissue section from this organ was stained 7 days after injection showing a significant decrease of the presence of gold with respect to 24 and 48 hours post-injection (Figure S8A). The aorta was the only tissue that did not stain positive for gold.


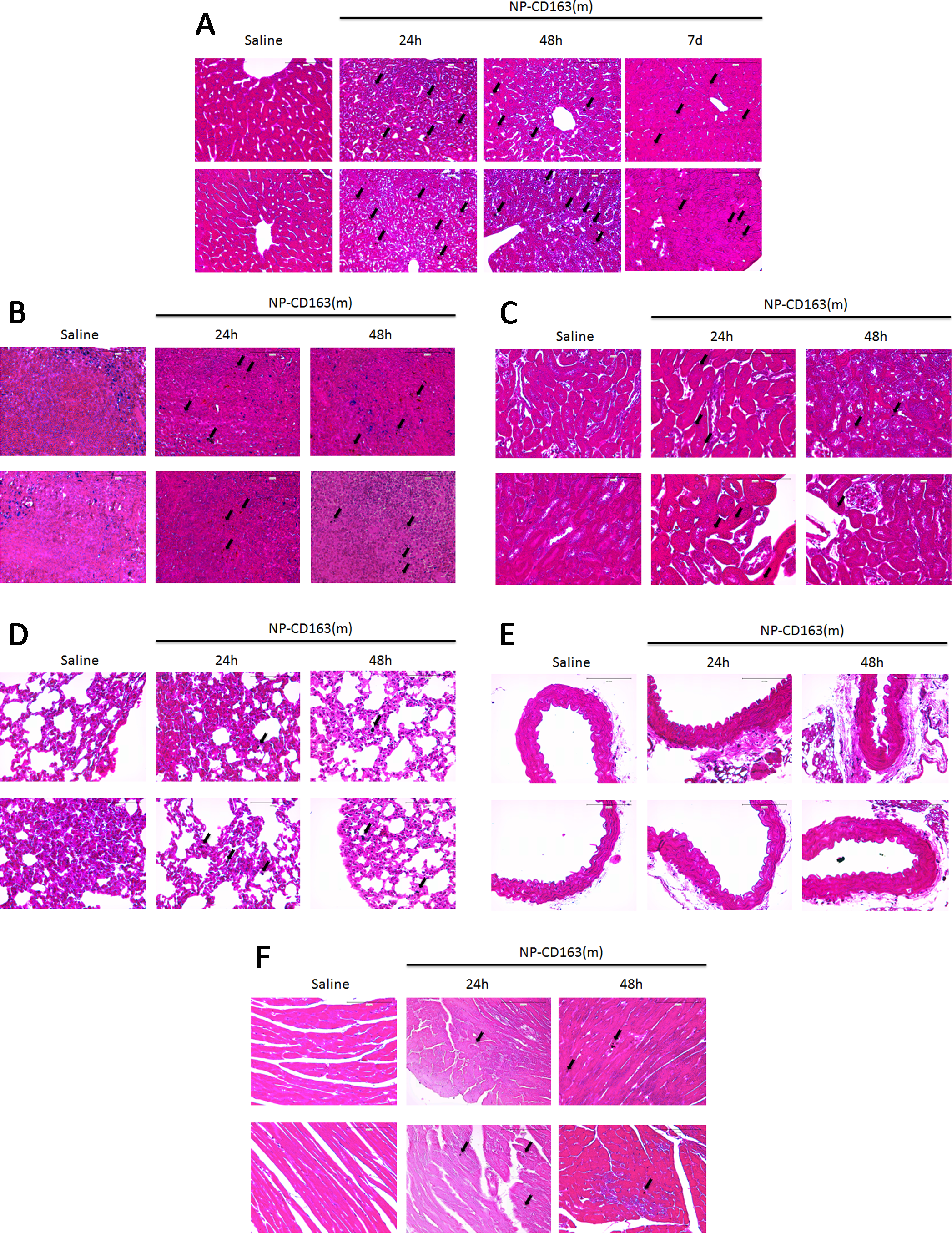


**Figure S8**. Gold staining to detect NP presence in tissue sections from different organs obtained from control mice injected with saline and mice at several time points after being injected with NP-CD163 (m). Black arrows show gold deposits. (A) Tissue sections from liver. (B) Tissue sections from spleen. (C) Tissue sections from kidney. (D) Tissue sections from lung. (E) Tissue sections from aorta. (F) Tissue sections from heart.

**7. Clearance of NPs from abdominal aortic wall after 6 days.**


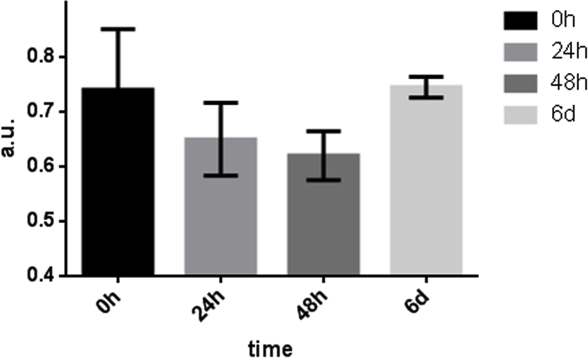


**Figure S9.** Comparison of the normalized contrast to noise ratio on the aortic wall of apoe-/- mice injected with NP-CD163(m) at different time points (0. 24. 48 hours and 6 days). The graph showed that after 6 days the signal in the aortic wall reached the pre-injection values.


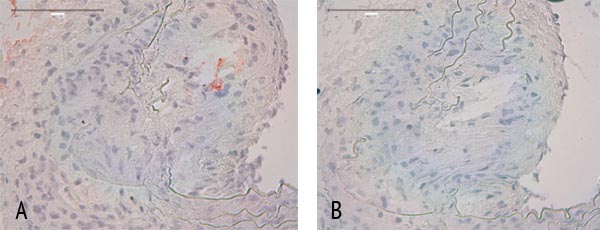


**Figure S10.** Immunohistochemistry with isotype-matched immunoglobulin as a negative control for CD163 (A) and CD68 (B).

| Levene’s test | df1 | df2 | Sig. |
| --- | --- | --- | --- |
| 4.475 | 6 | 14 | 0.01 |

**Table S2**.- Homogeneity variance test of THP-1 *in vitro* experiment.

| **One-way ANOVA** | | | | | |
| --- | --- | --- | --- | --- | --- |
|  | Sum of Squares | df | Mean Square | F | Sig. |
| Between Groups | 1.143 | 6 | 0.191 | 59.898 | 0.000 |
| Within Groups | 0.045 | 14 | 0.003 |  |  |
| Total | 1.188 | 20 |  |  |  |

**Table S3.**- One-way ANOVA test of THP-1 *in vitro* experiment.

| (I) Group | (J) Group | | Mean Diff. (I-J) | Std. Error | Sig. | 95% CI | |
| --- | --- | --- | --- | --- | --- | --- | --- |
|  |  |  |  |  |  | Lower Bound | Upper Bound |
| Blank | | NP-CD163 (-) | 0.150 | 0.047 | 0.310 | -0.266 | 0.566 |
|  |  | NP-CD163 (+) | 0.753* | 0.017 | 0.002 | 0.607 | 0.900 |
|  |  | NP-IgG (-) | 0.077 | 0.018 | 0.185 | -0.078 | 0.232 |
|  |  | NP-IgG (+) | 0.323 | 0.052 | 0.096 | -0.132 | 0.778 |
|  |  | Ab 1:1 + NP-CD163 (+) | 0.177 | 0.042 | 0.193 | -0.191 | 0.544 |
|  |  | Ab 1:10 + NP-CD163 (+) | 0.117* | 0.013 | 0.050 | -0.001 | 0.234 |
| NP-CD163 (-) | | Blank | -0.150 | 0.047 | 0.310 | -0.566 | 0.266 |
|  |  | NP-CD163 (+) | 0.603* | 0.050 | 0.014 | 0.253 | 0.954 |
|  |  | NP-IgG (-) | -0.073 | 0.050 | 0.761 | -0.419 | 0.273 |
|  |  | NP-IgG (+) | 0.173 | 0.070 | 0.353 | -0.178 | 0.524 |
|  |  | Ab 1:1 + NP-CD163 (+) | 0.027 | 0.063 | 0.999 | -0.291 | 0.344 |
|  |  | Ab 1:10 + NP-CD163 (+) | -0.033 | 0.049 | 0.983 | -0.402 | 0.335 |
| NP-CD163 (+) | | Blank | -0.753* | 0.017 | 0.002 | -0.900 | -0.607 |
|  |  | NP-CD163 (-) | -0.603* | 0.050 | 0.014 | -0.954 | -0.253 |
|  |  | NP-IgG (-) | -0.677* | 0.024 | 0.000 | -0.798 | -0.555 |
|  |  | NP-IgG (+) | -0.430* | 0.054 | 0.041 | -0.822 | -0.038 |
|  |  | Ab 1:1 + NP-CD163 (+) | -0.577* | 0.045 | 0.010 | -0.877 | -0.276 |
|  |  | Ab 1:10 + NP-CD163 (+) | -0.637* | 0.021 | 0.000 | -0.746 | -0.527 |
| NP-IgG (-) | | Blank | -0.077 | 0.018 | 0.185 | -0.232 | 0.078 |
|  |  | NP-CD163 (-) | 0.073 | 0.050 | 0.761 | -0.273 | 0.419 |
|  |  | NP-CD163 (+) | 0.677* | 0.024 | 0.000 | 0.555 | 0.798 |
|  |  | NP-IgG (+) | 0.247 | 0.055 | 0.136 | -0.140 | 0.634 |
|  |  | Ab 1:1 + NP-CD163 (+) | 0.100 | 0.045 | 0.480 | -0.196 | 0.396 |
|  |  | Ab 1:10 + NP-CD163 (+) | 0.040 | 0.022 | 0.603 | -0.075 | 0.155 |
| NP-IgG (+) | | Blank | -0.323 | 0.052 | 0.096 | -0.778 | 0.132 |
|  |  | NP-CD163 (-) | -0.173 | 0.070 | 0.353 | -0.524 | 0.178 |
|  |  | NP-CD163 (+) | 0.430* | 0.054 | 0.041 | 0.038 | 0.822 |
|  |  | NP-IgG (-) | -0.247 | 0.055 | 0.136 | -0.634 | 0.140 |
|  |  | Ab 1:1 + NP-CD163 (+) | -0.147 | 0.067 | 0.444 | -0.487 | 0.193 |
|  |  | Ab 1:10 + NP-CD163 (+) | -0.207 | 0.053 | 0.202 | -0.617 | 0.203 |
| Ab 1:1 + NP-CD163 (+) | | Blank | -0.177 | 0.042 | 0.193 | -0.544 | 0.191 |
|  |  | NP-CD163 (-) | -0.027 | 0.063 | 0.999 | -0.344 | 0.291 |
|  |  | NP-CD163 (+) | 0.577* | 0.045 | 0.010 | 0.276 | 0.877 |
|  |  | NP-IgG (-) | -0.100 | 0.045 | 0.480 | -0.396 | 0.196 |
|  |  | NP-IgG (+) | 0.147 | 0.067 | 0.444 | -0.193 | 0.487 |
|  |  | Ab 1:10 + NP-CD163 (+) | -0.060 | 0.044 | 0.795 | -0.377 | 0.257 |
| Ab 1:10 + NP-CD163 (+) | | Blank | -0.117* | 0.013 | 0.050 | -0.234 | 0.001 |
|  |  | NP-CD163 (-) | 0.033 | 0.049 | 0.983 | -0.335 | 0.402 |
|  |  | NP-CD163 (+) | 0.637* | 0.021 | 0.000 | 0.527 | 0.746 |
|  |  | NP-IgG (-) | -0.040 | 0.022 | 0.603 | -0.155 | 0.075 |
|  |  | NP-IgG (+) | 0.207 | 0.053 | 0.202 | -0.203 | 0.617 |
|  |  | Ab 1:1 + NP-CD163 (+) | 0.060 | 0.044 | 0.795 | -0.257 | 0.377 |
| *. The mean difference is significant at the 0.05 level | | | | | | | |

**Table S4**.- Games-Howel´s post hoc test of THP-1 *in vitro* experiment.

| Levene’s test | df1 | df2 | Sig. |
| --- | --- | --- | --- |
| 4.714 | 6 | 14 | 0.008 |

**Table S5**.- Homogeneity variance test of Murine macrophages *in vitro* experiment.

| **One-way ANOVA** | | | | | |
| --- | --- | --- | --- | --- | --- |
|  | Sum of Squares | df | Mean Square | F | Sig. |
| Between Groups | 0.803 | 6 | 0.134 | 40.767 | 0.000 |
| Within Groups | 0.046 | 14 | 0.003 |  |  |
| Total | 0.848 | 20 |  |  |  |

**Table S6**.- One-way ANOVA test of Murine macrophages *in vitro* experiment.

| (I) Group | (J) Group | | Mean Diff. (I-J) | Std. Error | Sig. | 95% CI | |
| --- | --- | --- | --- | --- | --- | --- | --- |
|  |  |  |  |  |  | Lower Bound | Upper Bound |
| Blank | | NP-CD163 (-) | 0.110 | 0.072 | 0.737 | -0.524 | 0.744 |
|  |  | NP-CD163 (+) | 0.643* | 0.030 | 0.008 | 0.383 | 0.904 |
|  |  | NP-IgG (-) | 0.140 | 0.029 | 0.152 | -0.114 | 0.394 |
|  |  | NP-IgG (+) | 0.187 | 0.024 | 0.063 | -0.025 | 0.398 |
|  |  | Ab 1:1 + NP-CD163 (+) | 0.180* | 0.006 | 0.004 | 0.129 | 0.231 |
|  |  | Ab 1:10 + NP-CD163 (+) | 0.060 | 0.012 | 0.134 | -0.042 | 0.162 |
| NP-CD163 (-) | | Blank | -0.110 | 0.072 | 0.737 | -0.744 | 0.524 |
|  |  | NP-CD163 (+) | 0.533* | 0.078 | 0.046 | 0.018 | 1.048 |
|  |  | NP-IgG (-) | 0.030 | 0.078 | 0.999 | -0.489 | 0.549 |
|  |  | NP-IgG (+) | 0.077 | 0.076 | 0.918 | -0.466 | 0.619 |
|  |  | Ab 1:1 + NP-CD163 (+) | 0.070 | 0.072 | 0.926 | -0.557 | 0.697 |
|  |  | Ab 1:10 + NP-CD163 (+) | -0.050 | 0.073 | 0.981 | -0.656 | 0.556 |
| NP-CD163 (+) | | Blank | -0.633* | 0.030 | 0.008 | -0.904 | -0.383 |
|  |  | NP-CD163 (-) | -0.533* | 0.078 | 0.046 | -1.048 | -0.018 |
|  |  | NP-IgG (-) | -0.503* | 0.041 | 0.002 | -0.710 | -0.297 |
|  |  | NP-IgG (+) | -0'.457* | 0.038 | 0.002 | -0.651 | -0.262 |
|  |  | Ab 1:1 + NP-CD163 (+) | -0.463* | 0.030 | 0.013 | -0.708 | -0.219 |
|  |  | Ab 1:10 + NP-CD163 (+) | -0.583* | 0.032 | 0.004 | -0.798 | -0.369 |
| NP-IgG (-) | | Blank | -0.140 | 0.029 | 0.152 | -0.394 | 0.114 |
|  |  | NP-CD163 (-) | -0.030 | 0.078 | 0.999 | -0.549 | 0.489 |
|  |  | NP-CD163 (+) | 0.503* | 0.041 | 0.002 | 0.297 | 0.710 |
|  |  | NP-IgG (+) | 0.047 | 0.038 | 0.849 | -0.144 | 0.237 |
|  |  | Ab 1:1 + NP-CD163 (+) | 0.040 | 0.029 | 0.798 | -0.197 | 0.277 |
|  |  | Ab 1:10 + NP-CD163 (+) | -0.080 | 0.031 | 0.381 | -0.288 | 0.128 |
| NP-IgG (+) | | Blank | -0.187 | 0.024 | 0.063 | -0.398 | 0.025 |
|  |  | NP-CD163 (-) | -0.077 | 0.076 | 0.918 | -0.619 | 0.466 |
|  |  | NP-CD163 (+) | 0.457* | 0.038 | 0.002 | 0.262 | 0.651 |
|  |  | NP-IgG (-) | -0.047 | 0.038 | 0.849 | -0.237 | 0.144 |
|  |  | Ab 1:1 + NP-CD163 (+) | -0.007 | 0.025 | 1.000 | -0.199 | 0.186 |
|  |  | Ab 1:10 + NP-CD163 (+) | -0.127 | 0.027 | 0.098 | -0.292 | 0.038 |
| Ab 1:1 + NP-CD163 (+) | | Blank | -0.180* | 0.006 | 0.004 | -0.231 | -0.129 |
|  |  | NP-CD163 (-) | -0.070 | 0.072 | 0.926 | -0.697 | 0.557 |
|  |  | NP-CD163 (+) | 0.463* | 0.030 | 0.013 | 0.219 | 0.708 |
|  |  | NP-IgG (-) | -0.040 | 0.029 | 0.798 | -0.277 | 0.197 |
|  |  | NP-IgG (+) | 0.007 | 0.025 | 1.000 | -0.186 | 0.199 |
|  |  | Ab 1:10 + NP-CD163 (+) | -0.120* | 0.013 | 0.016 | -0.199 | -0.041 |
| Ab 1:10 + NP-CD163 (+) | | Blank | -0.060 | 0.012 | 0.134 | -0.162 | 0.042 |
|  |  | NP-CD163 (-) | 0.050 | 0.073 | 0.981 | -0.556 | 0.656 |
|  |  | NP-CD163 (+) | 0.583* | 0.032 | 0.004 | 0.369 | 0.798 |
|  |  | NP-IgG (-) | 0.080 | 0.031 | 0.381 | -0.128 | 0.288 |
|  |  | NP-IgG (+) | 0.127 | 0.027 | 0.098 | -0.038 | 0.292 |
|  |  | Ab 1:1 + NP-CD163 (+) | 0.120* | 0.013 | 0.016 | 0.041 | 0.199 |
| *. The mean difference is significant at the 0.05 level | | | | | | | |

**Table S7**.- Games-Howel´s post hoc test of Murine macrophages *in vitro* experiment.

| Levene’s test | df1 | df2 | Sig. |
| --- | --- | --- | --- |
| 1.017 | 11 | 53 | 0.445 |

**Table S8**.- Homogeneity variance test of *in vivo* experiment.

| **One-way ANOVA** | | | | | |
| --- | --- | --- | --- | --- | --- |
|  | Sum of Squares | df | Mean Square | F | Sig. |
| Between Groups | 0.167 | 11 | 0.015 | 2.467 | 0.014 |
| Within Groups | 0.327 | 53 | 0.006 |  |  |
| Total | 0.494 | 64 |  |  |  |

**Table S9**.- One-way ANOVA test of *in vivo* experiment.

| (I) Group | (J) Group | Mean Diff. (I-J) | Std. Error | Sig. | 95% CI | |
| --- | --- | --- | --- | --- | --- | --- |
|  |  |  |  |  | Lower Bound | Upper Bound |
| ApoE-CD163 t=0 | ApoE-CD163 t=24h | 0.000 | 0.037 | 1.000 | -0.074 | 0.074 |
|  | ApoE-CD163 t=48h | 0.120* | 0.038 | 0.003 | 0.044 | 0.197 |
|  | ApoE-IgG t=0 | -0.054 | 0.044 | 0.223 | -0.142 | 0.034 |
|  | ApoE-IgG t=24h | -0.070 | 0.044 | 0.116 | -0.158 | 0.018 |
|  | ApoE-IgG t=48h | -0.024 | 0.044 | 0.586 | -0.112 | 0.064 |
|  | Wt-CD163 t=0 | -0.022 | 0.044 | 0.618 | -0.110 | 0.066 |
|  | Wt-CD163 t=24h | -0.010 | 0.044 | 0.820 | -0.098 | 0.078 |
|  | Wt-CD163 t=48h | -0.002 | 0.044 | 0.964 | -0.090 | 0.086 |
|  | Wt-IgG t=0 | -0.040 | 0.052 | 0.448 | -0.145 | 0.065 |
|  | Wt-IgG t=24h | -0.027 | 0.052 | 0.613 | -0.132 | 0.078 |
|  | Wt-IgG t=48h | 0.013 | 0.052 | 0.800 | -0.092 | 0.118 |
| ApoE-CD163 t=24h | ApoE-CD163 t=0 | 0.000 | 0.037 | 1.000 | -0.074 | 0.074 |
|  | ApoE-CD163 t=48h | 0.120* | 0.038 | 0.003 | 0.044 | 0.197 |
|  | ApoE-IgG t=0 | -0.054 | 0.044 | 0.223 | -0.142 | 0.034 |
|  | ApoE-IgG t=24h | -0.070 | 0.044 | 0.116 | -0.158 | 0.018 |
|  | ApoE-IgG t=48h | -0.024 | 0.044 | 0.586 | -0.112 | 0.064 |
|  | Wt-CD163 t=0 | -0.022 | 0.044 | 0.618 | -0.110 | 0.066 |
|  | Wt-CD163 t=24h | -0.010 | 0.044 | 0.820 | -0.098 | 0.078 |
|  | Wt-CD163 t=48h | -0.002 | 0.044 | 0.964 | -0.090 | 0.086 |
|  | Wt-IgG t=0 | -0.040 | 0.052 | 0.448 | -0.145 | 0.065 |
|  | Wt-IgG t=24h | -0.027 | 0.052 | 0.613 | -0.132 | 0.078 |
|  | Wt-IgG t=48h | 0.013 | 0.052 | 0.800 | -0.092 | 0.118 |
| ApoE-CD163 t=48h | ApoE-CD163 t=0 | -0.120* | 0.038 | 0.003 | -0.197 | -0.044 |
|  | ApoE-CD163 t=24h | -0.120* | 0.038 | 0.003 | -0.197 | -0.044 |
|  | ApoE-IgG t=0 | -0.174* | 0.045 | 0.000 | -0.264 | -0.084 |
|  | ApoE-IgG t=24h | -0.190* | 0.045 | 0.000 | -0.280 | -0.100 |
|  | ApoE-IgG t=48h | -0.144* | 0.045 | 0.002 | -0.234 | -0.054 |
|  | Wt-CD163 t=0 | -0.142* | 0.045 | 0.003 | -0.232 | -0.052 |
|  | Wt-CD163 t=24h | -0.130* | 0.045 | 0.005 | -0.220 | -0.040 |
|  | Wt-CD163 t=48h | -0.122* | 0.045 | 0.009 | -0.212 | -0.032 |
|  | Wt-IgG t=0 | -0.160* | 0.053 | 0.004 | -0.267 | -0.053 |
|  | Wt-IgG t=24h | -0.147* | 0.053 | 0.008 | -0.253 | -0.040 |
|  | Wt-IgG t=48h | -.107* | 0.053 | 0.050 | -0.213 | 0.000 |
| ApoE-IgG t=0 | ApoE-CD163 t=0 | 0.054 | 0.044 | 0.223 | -0.034 | 0.142 |
|  | ApoE-CD163 t=24h | 0.054 | 0.044 | 0.223 | -0.034 | 0.142 |
|  | ApoE-CD163 t=48h | 0.170* | 0.045 | 0.000 | 0.084 | 0.264 |
|  | ApoE-IgG t=24h | -0.016 | 0.050 | 0.749 | -0.116 | 0.084 |
|  | ApoE-IgG t=48h | 0.030 | 0.050 | 0.548 | -0.070 | 0.130 |
|  | Wt-CD163 t=0 | 0.032 | 0.050 | 0.522 | -0.068 | 0.132 |
|  | Wt-CD163 t=24h | 0.044 | 0.050 | 0.380 | -0.056 | 0.144 |
|  | Wt-CD163 t=48h | 0.052 | 0.050 | 0.300 | -0.048 | 0.152 |
|  | Wt-IgG t=0 | 0.014 | 0.057 | 0.808 | -0.101 | 0.129 |
|  | Wt-IgG t=24h | 0.027 | 0.057 | 0.636 | -0.088 | 0.142 |
|  | Wt-IgG t=48h | 0.067 | 0.057 | 0.246 | -0.048 | 0.182 |
| ApoE-IgG t=24h | ApoE-CD163 t=0 | 0.070 | 0.044 | 0.116 | -0.018 | 0.158 |
|  | ApoE-CD163 t=24h | 0.070 | 0.044 | 0.116 | -0.018 | 0.158 |
|  | ApoE-CD163 t=48h | 0.190* | 0.045 | 0.000 | 0.100 | 0.280 |
|  | ApoE-IgG t=0 | 0.016 | 0.050 | 0.749 | -0.084 | 0.116 |
|  | ApoE-IgG t=48h | 0.046 | 0.050 | 0.359 | -0.054 | 0.146 |
|  | Wt-CD163 t=0 | 0.048 | 0.050 | 0.338 | -0.052 | 0.148 |
|  | Wt-CD163 t=24h | 0.060 | 0.050 | 0.232 | -0.040 | 0.160 |
|  | Wt-CD163 t=48h | 0.068 | 0.050 | 0.177 | -0.032 | 0.168 |
|  | Wt-IgG t=0 | 0.030 | 0.057 | 0.603 | -0.085 | 0.145 |
|  | Wt-IgG t=24h | 0.043 | 0.057 | 0.453 | -0.072 | 0.158 |
|  | Wt-IgG t=48h | 0.083 | 0.057 | 0.152 | -0.032 | 0.198 |
| ApoE-IgG t=48h | ApoE-CD163 t=0 | 0.024 | 0.044 | 0.586 | -0.064 | 0.112 |
|  | ApoE-CD163 t=24h | 0.024 | 0.044 | 0.586 | -0.064 | 0.112 |
|  | ApoE-CD163 t=48h | 0.144* | 0.045 | 0.002 | 0.054 | 0.234 |
|  | ApoE-IgG t=0 | -0.030 | 0.050 | 0.548 | -0.130 | 0.070 |
|  | ApoE-IgG t=24h | -0.046 | 0.050 | 0.359 | -0.146 | 0.054 |
|  | Wt-CD163 t=0 | 0.002 | 0.050 | 0.968 | -0.098 | 0.102 |
|  | Wt-CD163 t=24h | 0.014 | 0.050 | 0.779 | -0.086 | 0.114 |
|  | Wt-CD163 t=48h | 0.022 | 0.050 | 0.660 | -0.078 | 0.122 |
|  | Wt-IgG t=0 | -0.016 | 0.057 | 0.781 | -0.131 | 0.099 |
|  | Wt-IgG t=24h | -0.003 | 0.057 | 0.963 | -0.118 | 0.112 |
|  | Wt-IgG t=48h | 0.037 | 0.057 | 0.518 | -0.078 | 0.152 |
| Wt-CD163 t=0 | ApoE-CD163 t=0 | 0.022 | 0.044 | 0.618 | -0.066 | 0.110 |
|  | ApoE-CD163 t=24h | 0.022 | 0.044 | 0.618 | -0.066 | 0.110 |
|  | ApoE-CD163 t=48h | 0.142* | 0.045 | 0.003 | 0.052 | 0.232 |
|  | ApoE-IgG t=0 | -0.032 | 0.050 | 0.522 | -0.132 | 0.068 |
|  | ApoE-IgG t=24h | -0.048 | 0.050 | 0.338 | -0.148 | 0.052 |
|  | ApoE-IgG t=48h | -0.002 | 0.050 | 0.968 | -0.102 | 0.098 |
|  | Wt-CD163 t=24h | 0.012 | 0.050 | 0.810 | -0.088 | 0.112 |
|  | Wt-CD163 t=48h | 0.020 | 0.050 | 0.689 | -0.080 | 0.120 |
|  | Wt-IgG t=0 | -0.018 | 0.057 | 0.755 | -0.133 | 0.097 |
|  | Wt-IgG t=24h | -0.005 | 0.057 | 0.935 | -0.120 | 0.110 |
|  | Wt-IgG t=48h | 0.035 | 0.057 | 0.541 | -0.080 | 0.150 |
| Wt-CD163 t=24h | ApoE-CD163 t=0 | 0.010 | 0.044 | 0.820 | -0.078 | 0.098 |
|  | ApoE-CD163 t=24h | 0.010 | 0.044 | 0.820 | -0.078 | 0.098 |
|  | ApoE-CD163 t=48h | 0.130* | 0.045 | 0.005 | 0.040 | 0.220 |
|  | ApoE-IgG t=0 | -0.044 | 0.050 | 0.380 | -0.144 | 0.056 |
|  | ApoE-IgG t=24h | -0.060 | 0.050 | 0.232 | -0.160 | 0.040 |
|  | ApoE-IgG t=48h | -0.014 | 0.050 | 0.779 | -0.114 | 0.086 |
|  | Wt-CD163 t=0 | -0.012 | 0.050 | 0.810 | -0.112 | 0.088 |
|  | Wt-CD163 t=48h | 0.008 | 0.050 | 0.873 | -0.092 | 0.108 |
|  | Wt-IgG t=0 | -0.030 | 0.057 | 0.603 | -0.145 | 0.085 |
|  | Wt-IgG t=24h | -0.017 | 0.057 | 0.773 | -0.132 | 0.098 |
|  | Wt-IgG t=48h | 0.023 | 0.057 | 0.686 | -0.092 | 0.138 |
| Wt-CD163 t=48h | ApoE-CD163 t=0 | 0.002 | 0.044 | 0.964 | -0.086 | 0.090 |
|  | ApoE-CD163 t=24h | 0.002 | 0.044 | 0.964 | -0.086 | 0.090 |
|  | ApoE-CD163 t=48h | 0.122* | 0.045 | 0.009 | 0.032 | 0.212 |
|  | ApoE-IgG t=0 | -0.052 | 0.050 | 0.300 | -0.152 | 0.048 |
|  | ApoE-IgG t=24h | -0.068 | 0.050 | 0.177 | -0.168 | 0.032 |
|  | ApoE-IgG t=48h | -0.022 | 0.050 | 0.660 | -0.122 | 0.078 |
|  | Wt-CD163 t=0 | -0.020 | 0.050 | 0.689 | -0.120 | 0.080 |
|  | Wt-CD163 t=24h | -0.008 | 0.050 | 0.873 | -0.108 | 0.092 |
|  | Wt-IgG t=0 | -0.038 | 0.057 | 0.511 | -0.153 | 0.077 |
|  | Wt-IgG t=24h | -0.025 | 0.057 | 0.669 | -0.140 | 0.090 |
|  | Wt-IgG t=48h | 0.015 | 0.057 | 0.790 | -0.100 | 0.130 |
| Wt-IgG t=0 | ApoE-CD163 t=0 | 0.040 | 0.052 | 0.448 | -0.065 | 0.145 |
|  | ApoE-CD163 t=24h | 0.040 | 0.052 | 0.448 | -0.065 | 0.145 |
|  | ApoE-CD163 t=48h | 0.160* | 0.053 | 0.004 | 0.053 | 0.267 |
|  | ApoE-IgG t=0 | -0.014 | 0.057 | 0.808 | -0.129 | 0.101 |
|  | ApoE-IgG t=24h | -0.030 | 0.057 | 0.603 | -0.145 | 0.085 |
|  | ApoE-IgG t=48h | 0.016 | 0.057 | 0.781 | -0.099 | 0.131 |
|  | Wt-CD163 t=0 | 0.018 | 0.057 | 0.755 | -0.097 | 0.133 |
|  | Wt-CD163 t=24h | 0.030 | 0.057 | 0.603 | -0.085 | 0.145 |
|  | Wt-CD163 t=48h | 0.038 | 0.057 | 0.511 | -0.077 | 0.153 |
|  | Wt-IgG t=24h | 0.013 | 0.064 | 0.836 | -0.115 | 0.142 |
|  | Wt-IgG t=48h | 0.053 | 0.064 | 0.409 | -0.075 | 0.182 |
| Wt-IgG t=24h | ApoE-CD163 t=0 | 0.027 | 0.052 | 0.613 | -0.078 | 0.132 |
|  | ApoE-CD163 t=24h | 0.027 | 0.052 | 0.613 | -0.078 | 0.132 |
|  | ApoE-CD163 t=48h | 0.147* | 0.053 | 0.008 | 0.040 | 0.253 |
|  | ApoE-IgG t=0 | -0.027 | 0.057 | 0.636 | -0.142 | 0.088 |
|  | ApoE-IgG t=24h | -0.043 | 0.057 | 0.453 | -0.158 | 0.072 |
|  | ApoE-IgG t=48h | 0.003 | 0.057 | 0.963 | -0.112 | 0.118 |
|  | Wt-CD163 t=0 | 0.005 | 0.057 | 0.935 | -0.110 | 0.120 |
|  | Wt-CD163 t=24h | 0.017 | 0.057 | 0.773 | -0.098 | 0.132 |
|  | Wt-CD163 t=48h | 0.025 | 0.057 | 0.669 | -0.090 | 0.140 |
|  | Wt-IgG t=0 | -0.013 | 0.064 | 0.836 | -0.142 | 0.115 |
|  | Wt-IgG t=48h | 0.040 | 0.064 | 0.535 | -0.089 | 0.169 |
| Wt-IgG t=48h | ApoE-CD163 t=0 | -0.013 | 0.052 | 0.800 | -0.118 | 0.092 |
|  | ApoE-CD163 t=24h | -0.013 | 0.052 | 0.800 | -0.118 | 0.092 |
|  | ApoE-CD163 t=48h | 0.107* | 0.053 | 0.050 | 0.000 | 0.213 |
|  | ApoE-IgG t=0 | -0.067 | 0.057 | 0.246 | -0.182 | 0.048 |
|  | ApoE-IgG t=24h | -0.083 | 0.057 | 0.152 | -0.198 | 0.032 |
|  | ApoE-IgG t=48h | -0.037 | 0.057 | 0.518 | -0.152 | 0.078 |
|  | Wt-CD163 t=0 | -0.035 | 0.057 | 0.541 | -0.150 | 0.080 |
|  | Wt-CD163 t=24h | -0.023 | 0.057 | 0.686 | -0.138 | 0.092 |
|  | Wt-CD163 t=48h | -0.015 | 0.057 | 0.790 | -0.130 | 0.100 |
|  | Wt-IgG t=0 | -0.053 | 0.064 | 0.409 | -0.182 | 0.075 |
|  | Wt-IgG t=48h | -0.040 | 0.064 | 0.535 | -0.169 | 0.089 |
| *. The mean difference is significant at the 0.05 level | | | | | | |

**Table S10**.- DMS post hoc test of *in vivo* experiment

1. Garcia. I.. et al.. *Magnetic Glyconanoparticles as a Versatile Platform for Selective Immunolabeling and Imaging of Cells.* Bioconjugate Chemistry. 2011. **22**(2): p. 264-273.

2. Gallo. J.. et al.. *Water-soluble magnetic glyconanoparticles based on metal-doped ferrites coated with gold: Synthesis and characterization.* Journal of Materials Chemistry. 2010. **20**(44): p. 10010-10020.

3. Moulder. J.F.. et al.. *Handbook of X‑ray Photoelectron Spectroscopy. Physical Electronics.* 1995.

4. Descostes. M.. et al.. *Use of XPS in the determination of chemical environment and oxidation state of iron and sulfur samples: constitution of a data basis in binding energies for Fe and S reference compounds and applications to the evidence of surface species of an oxidized pyrite in a carbonate medium.* Applied Surface Science. 2000. **165**(4): p. 288-302.
